# Eating Problems Among Adolescent Boys and Girls Before and During the Covid‐19 Pandemic

**DOI:** 10.1002/eat.24314

**Published:** 2024-10-30

**Authors:** Johanne H. Pettersen, Laura Hegemann, Kristin Gustavson, Ingunn Olea Lund, Pia Jensen, Cynthia M. Bulik, Ole A. Andreassen, Alexandra Havdahl, Ragnhild E. Brandlistuen, Laurie Hannigan, Helga Ask

**Affiliations:** ^1^ Department of Psychology University of Oslo Oslo Norway; ^2^ PsychGen Center for Genetic Epidemiology and Mental Health Norwegian Institute of Public Health Oslo Norway; ^3^ Department of Child Health and Development Norwegian Institute of Public Health Oslo Norway; ^4^ Nic Waals Institute Lovisenberg Diaconal Hospital Oslo Norway; ^5^ Department of Children and Families Norwegian Institute of Public Health Oslo Norway; ^6^ Department of Psychiatry University of North Carolina at Chapel Hill Chapel Hill North Carolina USA; ^7^ Department of Medical Epidemiology and Biostatistics Karolinska Institutet Stockholm Sweden; ^8^ Department of Nutrition University of North Carolina at Chapel Hill Chapel Hill North Carolina USA; ^9^ Center for Precision Psychiatry, Division of Mental Health and Addiction Oslo University Hospital Oslo Norway; ^10^ Institute of Clinical Medicine University of Oslo Oslo Norway; ^11^ PROMENTA Research Center, Department of Psychology University of Oslo Oslo Norway; ^12^ Division of Mental and Physical Health Norwegian Institute of Public Health Oslo Norway; ^13^ The Norwegian Mother, Father, and Child Cohort Study (MoBa) Norwegian Institute of Public Health Oslo Norway; ^14^ Population Health Sciences, Bristol Medical School University of Bristol Bristol UK

**Keywords:** adolescents, Covid‐19, eating problems, genetics, MoBa, structural equation modeling

## Abstract

**Objective:**

Studies suggest that adolescents reported more eating problems during the pandemic. Using a population‐based sample, we compared eating problems—and how they associate with a range of personal characteristics and genetic factors—among adolescents before (June 2017–April 2020) versus during (April 2020–December 2022) the pandemic.

**Method:**

Based on a preregistered analysis plan, we used cross‐sectional data collected from 22,706 14–16‐year‐olds over 6 years (55% during the pandemic) in the Norwegian Mother, Father, and Child Cohort. We used measurement invariance analyses to compare the level of eating restraint and body concern before and during the pandemic, and multi‐group structural equation models to estimate pre‐pandemic and pandemic patterns of associations.

**Results:**

Pandemic responders generally reported more eating problems than pre‐pandemic responders, specifically on dieting and body dissatisfaction. However, after adjusting for a general linear increase in eating problems across all 6 years of data collection, the pandemic itself seems to be associated with more eating problems only among girls, reporting more eating restraints (meanΔ = 0.14 [CI: 0.07, 0.20]) and body concern (meanΔ = 0.17 [CI: 0.11, 0.23]). Associations between eating problems and a range of other characteristics did not differ across the pandemic and pre‐pandemic groups.

**Conclusions:**

There was a general increase in eating problems among 14–16‐year‐olds over time. Adjusting for this trend, the pandemic seems to exacerbate problems among girls. Although the mechanisms are unclear, our results point to factors susceptible to change that could have been intensified during the pandemic (e.g., screen time, mental distress). Our results highlight the importance of recognizing sex‐specific differences in eating problems.

**Summary**

- This study highlights a general increase in eating problems among adolescents over time, with the Covid‐19 pandemic potentially worsening problems among girls.
- We emphasize the need to address sex‐specific factors in the understanding and treatment of eating problems.
- Increased screen time, mental distress, stress, exercise and parent–child conflict during the pandemic may have contributed to these problems, highlighting areas for targeted interventions to improve adolescent well‐being.

## 1 Introduction

The onset of the Covid‐19 pandemic resulted in disruption of routines, social isolation, and increased mental distress among adolescents (Racine et al. 2021; Hawes et al. 2021; Panchal et al. 2021), and represents an opportunity to identify psychosocial factors involved in the etiology of mental illness (Unnarsdóttir et al. 2022). The Norwegian government implemented several public health measures, including stay‐at‐home mandates, school closures, and travel restrictions during the pandemic, with the highest stringency levels occurring from March to May 2020 and January to May 2021 (Government.no n.d.). Beyond an apparently increasing trend of eating disorders (Galmiche et al. 2019) and problems in general (von Soest and Wichstrøm 2014), a drastic increase in eating disorders has been reported among adolescents, particularly girls during the pandemic (Otto et al. 2021; Surén et al. 2022; Silén and Keski‐Rahkonen 2022; Cerniglia and Cimino 2022; Trafford et al. 2023; Taquet et al. 2022). For instance, in girls aged 13–16 years, the relative increase in reported eating disorder diagnoses was 127% and 96% in primary and specialist care, respectively (Surén et al. 2022). This increase represents both an intrinsically important public health issue, which may inform management of similar crises in the future, and a compelling natural experiment to explore the underlying mechanisms related to eating problems.

Adolescence brings social, physical, and emotional changes that may contribute to the development of eating problems (Mora et al. 2022; Smolak and Levine 2015). Eating problems reflect a spectrum of unhealthy eating behaviors and attitudes related to food and body image, including negative perceptions and feelings about one's own body, shape, or weight (Pereira and Alvarenga 2007). For some, such eating problems can escalate into more complex and severe conditions, collectively referred to as eating disorders. Anorexia nervosa (AN), bulimia nervosa, binge‐eating disorder, and other eating disorders are characterized by disturbances in eating behaviors and associated thoughts and emotions. Eating disorders typically develop around mid‐adolescence (Uhlhaas et al. 2023), with considerable variation across genders, impacting 6%–18% of girls and 2% of boys (Silén and Keski‐Rahkonen 2022).

Observational studies including subclinical eating problems before and during the pandemic have yielded mixed results (Schneider et al. 2023). A recent systematic review highlighted the variability in individuals reactions to the pandemic, with some studies reporting improvements in body image and disordered eating, while others indicated deterioration or no significant changes (Schneider et al. 2023). Eating problems among boys have been notably understudied. For girls, concerns about body weight and shape are typically related to achieving or maintaining thinness, whereas boys are more likely to strive for an ideal linked to muscularity and leanness (Hornberger et al. 2021; Gorrell and Murray 2019; Lavender, Brown, and Murray 2017; Neumark‐Sztainer et al. 2006; Murray et al. 2017). This highlights the importance of considering sex‐specific differences in symptoms. Behaviors associated with striving for these ideals could include excessive exercise, rigid dieting behaviors, binge eating or purging (Trafford et al. 2023; Taquet et al. 2022; Lavender, Brown, and Murray 2017; Neumark‐Sztainer et al. 2006).

Several studies have investigated risk factors associated with eating disorders and problems in adolescents, consistently reporting associations with factors such as depression, social media and smartphone use, poor family dynamics, stress, higher body‐mass index (BMI) (Suarez‐Albor, Galletta, and Gómez‐Bustamante 2022; Varela et al. 2023), and co‐occurring neurodevelopmental and mental health conditions, including anxiety, autism, and obsessive‐compulsive disorder (OCD) (Varela et al. 2023; Carpita et al. 2022). Research also highlights the genetic components of eating disorders, showing moderate heritability (Yilmaz, Hardaway, and Bulik 2015; Watson et al. 2019) and higher incidence among relatives of affected individuals (Strober et al. 2000; Lilenfeld et al. 1998). Furthermore, genetic liability to AN (Yilmaz et al. 2022), higher BMI and neuroticism has been linked to eating problems (Micali et al. 2015; Robinson et al. 2020). However, most of these studies have been limited to small, predominantly female samples, and few have examined how these associations vary over time, particularly in the context of crises like the Covid‐19 pandemic.

In this preregistered study, we used cross‐sectional questionnaire data collected from 22,706 14–16‐year‐olds over 3 years before (2017–2020) and 3 years during (2020–2022) the pandemic, enriched with data from national health registries and genetic information. Each adolescent reported on eating problems, either before or during the pandemic, providing a unique opportunity to investigate the following pre‐specified hypotheses (1) eating problems were more frequent during, compared to before the pandemic; (2) personal characteristics and genetic scores for mental health conditions were associated with eating problems among adolescent girls and boys; and (3) the strength of these associations changed with the onset of the pandemic.

## 2 Methods

### 2.1 Study Design and Sample

We used data from the Norwegian Mother, Father, and Child Cohort Study (MoBa), a population‐based pregnancy cohort study conducted by the Norwegian Institute of Public Health (Magnus et al. 2006, 2016). Pregnant women were recruited when attending a routine ultrasound examination across local hospitals all over Norway from 1999 to 2008. The women consented to participation in 41% of the pregnancies. The cohort includes approximately 114,500 children, 95,200 mothers, and 75,200 fathers. Blood samples were obtained from children (umbilical cord) at birth (Paltiel et al. 2014). Genotype data were quality‐controlled using the MoBaPsychGen pipeline (Corfield et al. 2022). Compared to the general population, MoBa participants are, on average, from more highly educated and healthier families than the general population. Specifically, the pregnant women who participated included fewer younger women (under 25 years old), those living alone, and smokers (Nilsen et al. 2009). Although we did not use any weighting to address these biases, the use of multiple imputation can account for some selection bias and attrition in MoBa (Nilsen et al. 2009; Biering, Hjollund, and Frydenberg 2015; Biele et al. 2019). The Norwegian Health Registry Act currently regulates the MoBa cohort. The current study was approved by the Regional Committees for Medical and Health Research (#14140). We linked MoBa data to data from the Norwegian Patient Registry (NPR), the Payment of Health Reimbursements Database (KUHR), the Medical Birth Registry of Norway, and Statistics Norway. NPR provides information on specialist healthcare use from 2008 to 2020, including the International Classification of Diseases (ICD‐10) (World Health Organization 1993) codes, and KUHR provides the International Classification of Primary Care (ICPC‐2) (Verbeke et al. 2006) codes from 2006 to 2020. Statistics Norway provided information on parents' education. This paper was preregistered in the open science framework (OSF): https://osf.io/2eqz5/. See Table S1 for a complete list of deviations from the preregistration.

Since 2017, adolescents in MoBa have been invited to answer a questionnaire the year they turned 14‐years old. A total of 22,706 adolescents (12,148 girls and 10,558 boys) born between 2002 and 2007 were included in our analyses. Adolescents who answered between June 2017 and April 12, 2020, were assigned to the pre‐pandemic group (5655 girls and 4675 boys), and adolescents who answered in the first 32 months after this date were categorized as the pandemic group (6493 girls and 5882 boys), see flow chart in Figure S1. The cut‐off date was selected because the adolescents were asked about their eating problems during the last 4 weeks, and the first national lockdown date in Norway was March 12, 2020. A higher number of adolescents responded to the questionnaire during the pandemic (12,375), compared to before the pandemic (10,330). However, of adolescents invited to respond, response rates dropped from 34.6% to 30.7% during the pandemic showing a significant difference in proportions, *χ* ^2^(1, *N* = 71,163) = 120.4, *p* < 0.001.

### 2.2 Eating Problems

The 14‐year questionnaire included 8 items from the Eating Disorder Examination Questionnaire (EDE‐Q) (Fairburn and Beglin 1994), selected from three subscales of food restraint, eating concern, and shape concern. All responses were scored from 0 to 3 (ranging from “Not at all”/“Never” to “Much”/“Often”). In addition to the EDE‐Q items, an item concerning how the adolescent considers their weight was included which was scored from 0 to 2 (“Okay”/“A little too thin/fat”/“Too thin/fat”). See Supporting Information: Section 1 for further description of items.

We randomly split the total sample in half and ran exploratory factor analysis (EFA) on one half to explore the underlying factor structure of the nine eating problem variables (Costello and Osborne 2019) (see Supporting Information: Section 2). The number of factors (Racine et al. 2021; Hawes et al. 2021; Panchal et al. 2021) was selected based on a comparison of model fit (i.e., comparative fit index [CFI], root mean square error of approximation [RMSEA]), standardized root mean square residual [SRMR], and parallel analysis methods using the *psych* package (Revelle 2015) (see Table S2 for fit measures). The EFA was run using a weighted least squares with mean and variance adjusted (WLSMV) estimator and Geomin oblique rotation in the *lavaan* package (Rosseel 2012). Because of the categorical nature of the responses, we estimated polychoric correlations. See Table S3 for factor loadings. Confirmatory factor analysis (CFA) for the best‐fitting EFA (a two‐factor structure) was run on the other half of the sample using WLSMV estimator in *lavaan* to estimate model fit. See Supporting Information: Section 3 and Table S4 for further details on CFA and model comparison, respectively.

Stepwise measurement invariance testing was run across sex to estimate whether further analyses needed to be sex‐stratified, revealing measurement differences in eating problems between girls and boys (see Supporting Information: Section 4 for a description of the steps, model fit criteria, and results). Table S5 shows the model fit comparison across measurement invariance steps. EFA/CFA (as described above) were therefore run separately for girls and boys revealing the same two‐factor structure (see Supporting Information: Section 5 for EFA/CFA results and Figure S2 for factor structure and factor loadings). Tables S6‐S8 shows EFA/CFA model comparisons and factor loadings. The first 5 items (Q1:Q5) loaded highest on a factor we called “Eating restraint” and the final 4 items (Q6:Q9) loaded highest on the second factor called “Body concern.”

### 2.3 Personal Characteristics

See Supporting Information: Section 6 for further details on the measurement, preparation, and definitions of predictors/covariates.

Exercise was measured by one item reporting the amount of time adolescents usually spent exercising during a week, scored from 0 (never) to 6 (11+ h). Social media communication and screen time measured the amount of time the adolescents usually spent on a weekday on these activities. The variables were scored from 1 (never) to 6 (7+ h).

The adolescents were classified as either “normal weight,” “underweight,” or “overweight” for their age and sex based on a Norwegian BMI calculator (Norsk Helseinformatikk (NHI) 2024). BMI was coded categorical to account for the possible nonlinear association between BMI and eating problems. Adolescents missing either height or weight values were set to missing.

Mental distress was measured by the Hopkins Symptom Checklist (SCL‐10) (Strand et al. 2003). SCL‐10 is scored on a 4‐point scale from 1 (not bothered) to 4 (very bothered). The four‐item Perceived Stress Scale (PSS‐4) (Cohen, Kamarck, and Mermelstein 1983) was utilized to measure perceived stress levels. The PSS‐4 items are scored on a 5‐point scale from 0 (never) to 4 (very often).

Conflict with parents was measured by 4 items from the Parent–Child Conflict Scale, originally comprised of 12 items (Elkins, McGue, and Iacono 1997). Conflict with parents was scored on a 4‐point scale from 1 (never) to 4 (almost all the time). Mother's history of eating disorder was coded “Yes” if the mothers answered yes to the question “Do you have or have you had anorexia/bulimia/other eating disorders” asked when they were 15‐weeks pregnant or if they received a diagnosis of an eating disorder in NPR (ICD‐10 code F50) or KUHR (ICPC‐2 code P86) before the adolescents answered the questionnaire, and otherwise was coded “No.”

Mean scores for mental distress, stress, and parent–child conflict were calculated based on the requirement of participants answering at least 50% of the items.

#### 2.3.1 Genetic Factors

Polygenic scores (PGS) are measures of an individual's genetic liability to a specific trait or condition. The PGS were created based on results from previous genome‐wide association studies on AN (Watson et al. 2019), anxiety (Purves et al. 2020), depression (Howard et al. 2019), neuroticism (Nagel et al. 2018), OCD (Arnold et al. 2018), BMI (Hammond et al. 2021), autism spectrum condition (autism) (Grove et al. 2019).

Adolescent's age and year of response (coded 0–2 within pre‐pandemic and pandemic groups) were added as continuous covariates in the SEM models to account for possible differences within the pre‐pandemic and pandemic groups due to age and cohort variations/time trends in eating problems.

### 2.4 Statistical Analyses

All our analyses were run using R 2021 and separately for girls and boys. Analysis code is publicly available on GitHub: https://github.com/psychgen/Covid19eatingproblems. To estimate whether adolescents reported more eating problems during the pandemic compared to before the pandemic, we first ran measurement invariance testing using multigroup CFA across pandemic groups (i.e., pre‐pandemic and pandemic) using the *lavaan* package (Rosseel 2012) (see Supporting Information: Section 7 for details). If strong invariance was achieved, differences in eating problems could be compared across the two groups. Next, we ran a multi‐group CFA and constrained factor means to be 0 in the pre‐pandemic group and estimated the factor means in the pandemic group (i.e., indicating the factor mean difference across the groups). Then, the same model was run with year of response included as a predictor to account for time trends within the groups.

Additionally, we used separate Chi‐square trend tests to quantify group differences in the observed frequency of the ordinal responses and investigate which of the nine eating problem items were more often reported over time/during the pandemic.

The associations between the selected predictors and eating restraint and body concern were estimated by adding them to the SEM model (adjusted for age and year of response). To investigate whether the associations between the predictors and the latent factors varied in strength according to whether eating problems were assessed before or during the pandemic, multi‐group SEM models were run, including pre‐pandemic/pandemic as a group variable. Associations between each predictor and the eating problem outcomes were first estimated in both groups and subsequently constrained to be equal across the two groups. Model fit of each constrained model and the unconstrained model were compared using the lavTestLRT.mi() function from the *semTools* (Jorgensen et al. 2022) package.

Multiple imputation was used to handle missing data in covariates. The *mice* package was used to create 50 imputed datasets and the runMI() function from the semTools package was used to pool the datasets using Rubin's rules (Rubin 1987). This differs from what was described in the preregistration, where full information maximum likelihood (FIML) was the anticipated approach. FIML is currently not an available option for the WLSMV optimizer in *lavaan* (Categorical data n.d.). The Benjamini‐Hochberg procedure (Benjamini and Hochberg 1995) was used to adjust for false discovery rate (FDR) due to multiple testing in the SEM and multi‐group SEM models. All estimates were corrected for two tests (i.e., two latent factors), and the BMI estimates were corrected for a total of four tests (i.e., two BMI dummy variables and two factors).

In post hoc analyses, we ran independent *t*‐tests and chi‐square tests to evaluate mean and distribution differences between pre‐pandemic and pandemic responses concerning predictors susceptible to change during or due to the pandemic, including exercise, social media communication, screen time, stress, mental distress, conflict with parents, and BMI.

As sensitivity analyses, we re‐ran the multigroup SEM on a sample excluding participants who answered the questionnaire within the 2 weeks before and after the cut‐off date of April 12th. This approach addresses possible ambiguities around the cut‐off date separating the pre‐pandemic from the pandemic group and aims to strengthen confidence in our results.

## 3 Results

Demographic characteristics of the girls and boys who were invited and participated before (June 2017–April 2020) and during (April 2020–December 2022) the pandemic are shown in Table 1. Participants were 14 (59%) or 15‐years old (40%) when responding to the questionnaire; the remainder were 16. Among adolescents who responded to the questionnaire, 83% had parents with at least a bachelor's degree, compared to 74% among all invited adolescents. However, the level of parental education was relatively similar across the pre‐pandemic and pandemic groups, with the majority of parents (80% pre‐pandemic and 79% during the pandemic) possessing at least a bachelor's degree. We identified a two‐factor solution (eating restraint and body concern) for girls and boys underlying eating problems.

**TABLE 1:** Sociodemographic characteristics.

Sociodemographic characteristics	Girls		Boys		Total sample		All invited adolescents	
	(*N* = 12,148)		(*N* = 10,558)		(*N* = 22,706)		(*N* = 71,163)	
	Pre‐pandemic (*N* = 5655)	Pandemic (*N* = 6493)	Pre‐pandemic (*N* = 4676)	Pandemic (*N* = 5882)	Pre‐pandemic (*N* = 10,331)	Pandemic (*N* = 12,375)	Pre‐pandemic (*N* = 30,707)	Pandemic (*N* = 40,456)
Age (years)								
14	3339 (59.0%)	3831 (59.0%)	2719 (58.2%)	3446 (58.6%)	6058 (58.6%)	7277 (58.8%)	9282 (30.2%)	7334 (18.1%)
15	2212 (39.1%)	2658 (40.9%)	1867 (39.9%)	2433 (41.4%)	4079 (39.5%)	5091 (41.1%)	19,642 (64.0%)	32,998 (81.6%)
16	104 (1.8%)	< 10	90 (1.9%)	< 10	194 (1.9%)	7 (0.1%)	1749 (5.7%)	35 (0.1%)
Year born								
2002	398 (7.0%)	< 10	308 (6.6%)	< 10	706 (6.8%)	< 10	2473 (8.1%)	< 10
2003	2270 (40.1%)	< 10	1979 (42.3%)	< 10	4249 (41.1%)	< 10	12,054 (39.3%)	< 10
2004	2308 (40.8%)	95 (1.5%)	1873 (40.1%)	104 (1.8%)	4181 (40.5%)	199 (1.6%)	12,935 (42.1%)	30 (0.1%)
2005	679 (12.0%)	2078 (32.0%)	516 (11.0%)	1833 (31.2%)	1195 (11.6%)	3911 (31.6%)	3211 (10.5%)	11,700 (28.9%)
2006	< 10	2927 (45.1%)	< 10	2661 (45.2%)	< 10	5588 (45.2%)	< 10	16,506 (40.8%)
2007	< 10	1393 (21.5%)	< 10	1283 (21.8%)	< 10	2676 (21.6%)	< 10	12,131 (30.0%)
Parents' education								
Compulsory	71 (1.3%)	54 (0.8%)	59 (1.3%)	38 (0.6%)	130 (1.3%)	92 (0.7%)	884 (2.9%)	907 (2.2%)
Upper secondary	1070 (18.9%)	963 (14.9%)	872 (18.6%)	787 (13.4%)	1942 (18.8%)	1750 (13.1%)	8229 (26.8)	8288 (20.5)
Bachelor's degree	2923 (51.7%)	3179 (49.0%)	2441 (52.2%)	2867 (48.7%)	5364 (51.9%)	6046 (45.2%)	14,939 (48.7)	19,436 (48.0%)
Master's degree or Ph.D.	1589 (28.1%)	2294 (35.3%)	1302 (27.8%)	2187 (37.2%)	2891 (28.0%)	4481 (33.5%)	6686 (21.8)	11,802 (29.2%)
Mother's history of eating disorder								
Yes	168 (3.0%)	189 (2.9%)	127 (2.7%)	221 (3.8%)	295 (2.9%)	410 (3.3%)	1002 (3.3%)	1415 (3.5%)
No	5487 (97.0%)	6304 (97.1%)	4549 (97.3%)	5661 (96.2%)	10,036 (97.1%)	11,965 (96.7%)	29,705 (96.7%)	39,041 (96.5)

*Note*: The table displays sociodemographic characteristics in count (%) for adolescents in the pre‐pandemic and pandemic groups.

### 3.1 Main Analyses

#### 3.1.1 Eating Problems Before Versus During the Pandemic

The establishment of complete measurement invariance between pandemic exposure groups (see Table S9) allowed the comparison of latent means of the two factors (eating restraint and body concern) in multi‐group CFA/SEM models. All confidence intervals (CIs) were calculated based on 95% confidence. Girls reported higher levels of eating restraint (mean difference expressed in standard deviations = 0.14 [CI: 0.10, 0.18]) and body concern (mean difference = 0.14 [CI: 0.11, 0.18]) during the pandemic, compared to girls reporting prior to the pandemic. Boys also reported higher levels of body concern (mean difference = 0.10 [CI: 0.05, 0.14]) during the pandemic. After adjusting for year of response (within pandemic groups), girls reported higher levels of both eating restraint (mean difference = 0.14 [CI: 0.07, 0.20]) and body concern (mean difference = 0.17 [CI: 0.11, 0.23]) during the pandemic, while for boys, eating problems did not differ significantly between the pandemic exposure groups after accounting for the general time trend.

**FIGURE 1:**
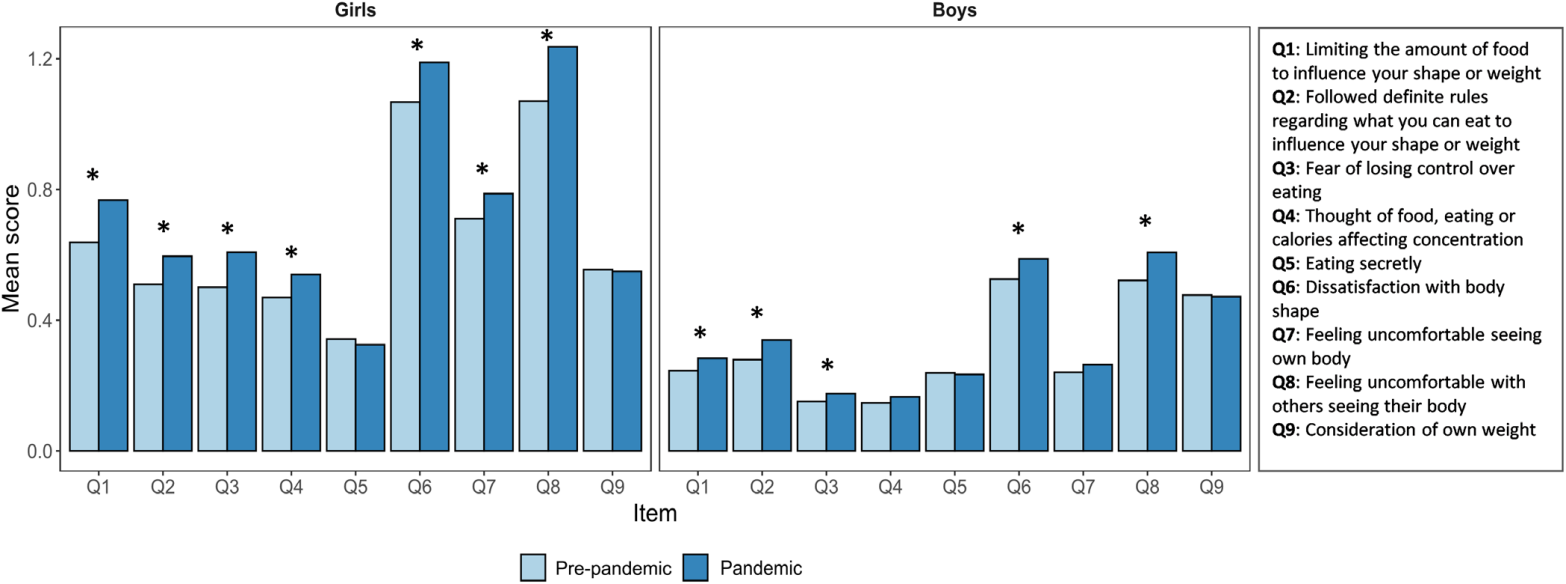
Mean score for each eating problem item across sex for pre‐pandemic and pandemic groups. *Note*: * = Significant chi‐square trends between pre‐pandemic and pandemic group.

The chi‐square trend tests for seven of the nine eating problem items in girls revealed a higher frequency of response categories indicating eating problems when responded to during the pandemic compared to before. The two items without higher frequency were: “eating secretly” and “consideration of own weight.” For boys, the following five items showed significantly higher trends during the pandemic: “limiting the amount of food to influence shape or weight,” “following definite rules regarding what you can eat to influence your shape or weight,” “fear of losing control over eating,” “dissatisfaction with body shape,” and “feeling uncomfortable seeing own body” (see results for all items in Table S10). Figure 1 shows the mean value for each item stratified by sex and pandemic exposure. Figure S3 shows the item means stratified by sex and year answered, showing higher means for most items over time.

#### 3.1.2 Personal Characteristics Associated With Eating Restraint

Standardized estimates for the cross‐sectional associations between the selected personal characteristics with adolescent eating problems are presented in Figure S4. For both boys and girls, eating restraint was positively associated with exercise (*β*_girls_ = 0.04 [CI: 0.02, 0.05], *β*_boys_ = 0.05 [0.04, 0.07]); social media communication (*β*_girls_ = 0.06 [0.04, 0.08], *β*_boys_ = 0.04 [0.02, 0.06]); being overweight (*β*_girls_ = 0.43 [0.38, 0.49], *β*_boys_ = 0.67 [0.61, 0.73]); mental distress (*β*_girls_ = 0.55 [0.51, 0.59], *β*_boys_ = 0.59 [0.52, 0.65]); perceived stress (*β*_girls_ = 0.28 [0.24, 0.31], *β*_boys_ = 0.23 [0.19, 0.27]); and conflict with parents (*β*_girls_ = 0.20 [0.16, 0.23], *β*_boys_ = 0.11 [0.05, 0.16]). Notably, for boys only, there was an association between mother's history of eating disorder (*β* = 0.15 [0.03, 0.26]) and eating restraint.

#### 3.1.3 Personal Characteristics Associated With Body Concern

Exercising was associated with less body concern (*β*_girls_ = −0.04 [−0.05, −0.03], *β*_boys_ = −0.07 [−0.08, −0.06]). Body concern was positively associated with being overweight (*β*_girls_ = 0.68 [0.62, 0.73], *β*_boys_ = 0.60 [0.55, 0.66]); mental distress (*β*_girls_ = 0.59 [0.55, 0.63], *β*_boys_ = 0.68 [0.62, 0.73]); perceived stress (*β*_girls_ = 0.37 [0.33, 0.40], *β*_boys_ = 0.27, CI: 0.23, 0.30); and conflict with parents (*β*_girls_ = 0.17 [0.13, 0.21], *β*_boys_ = 0.15 [0.10, 0.19]). Social media communication (*β*_girls_ = 0.04 [0.02, 0.06]) and screen time (*β*_girls_ = 0.04 [0.02, 0.06]) were significantly associated with more body concern in girls. For boys only, there was an association between being underweight and more body concern (*β* = 0.21 [0.12, 0.30]).

#### 3.1.4 Genetic Factors and Eating Problems

Figure S5 presents estimates for associations between PGS and eating problems. For both boys and girls, eating restraint was positively associated with genetic liability for high BMI (*β*_girls_ = 0.06 [0.04, 0.08], *β*_boys_ = 0.08 [0.06, 0.11]). In girls only, eating restraint was positively associated with genetic liability for AN (*β* = 0.02 [0.00, 0.04]) and neuroticism (*β* = 0.03 [0.01, 0.05]). Body concern was positively associated with genetic liability for neuroticism (*β*_girls_ = 0.04 [0.02, 0.06], *β*_boys_ = 0.04 [0.02, 0.06]), and with genetic liability for high BMI in girls only (*β* = 0.05 [0.03, 0.07]). All standardized estimates from our model can be found in Table S11.

#### 3.1.5 Associations With Eating Problems Before and During the Pandemic

**FIGURE 2:**
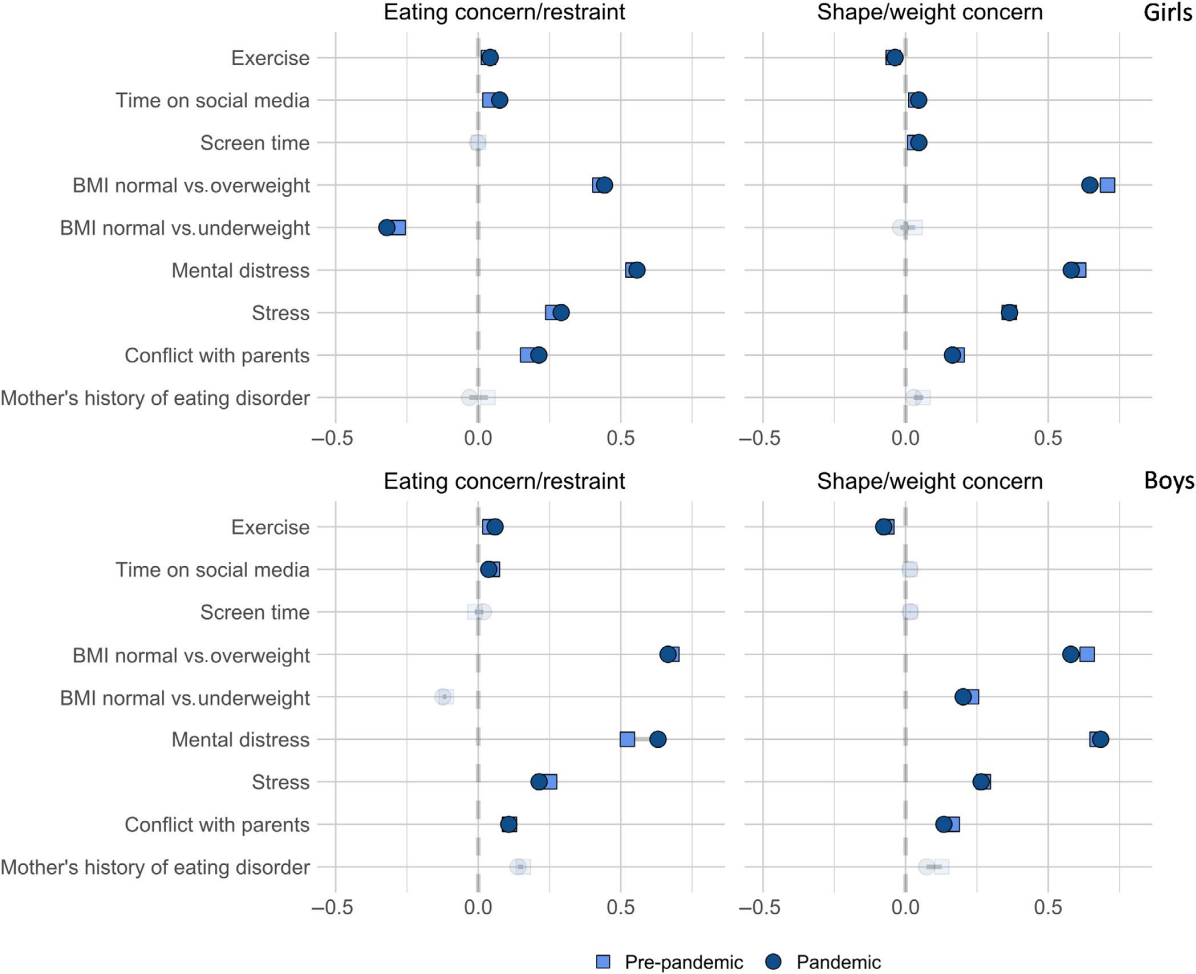
Standardized estimates from multi‐group SEM models of associations between predictors and eating problem domains in the pre‐pandemic and pandemic groups. *Note*: The transparent estimates indicate nonsignificant effects.

**FIGURE 3:**
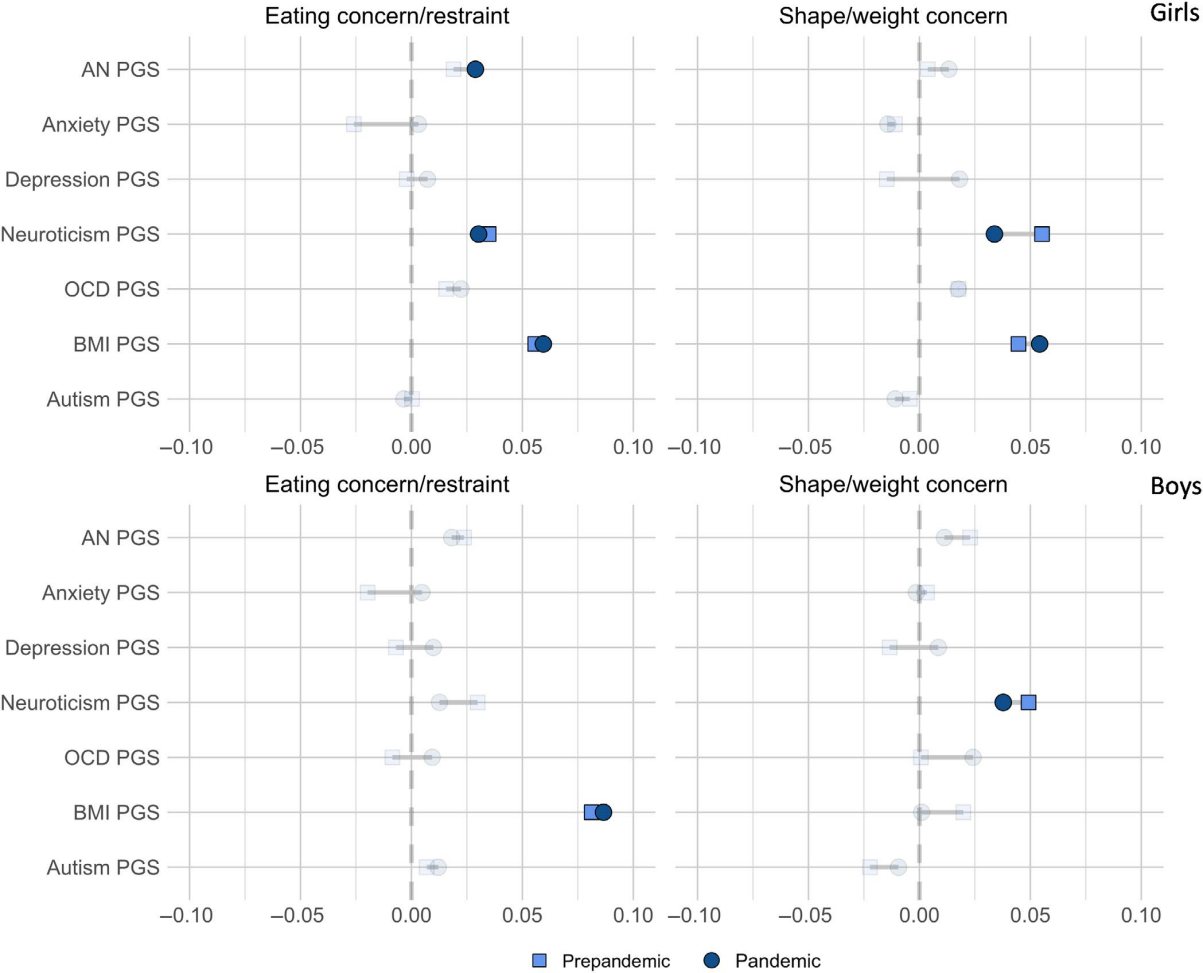
Standardized estimates from multi‐group SEM models of associations between PGS and eating problem domains in the pre‐pandemic and pandemic groups. *Note*: The transparent estimates indicate nonsignificant effects.

The associations between the selected variables and eating problems could be constrained to be similar across the pre‐pandemic and pandemic groups without significantly decreasing model fit, indicating no change in the associations between groups (see Figure 2 for personal characteristics and Figure 3 for PGS associations). Tables S12 and S13 show model fit comparisons between an unconstrained model and a model where each predictor is constrained to be equal across groups for girls and boys, respectively. Model fit indices for all SEM and multi‐group SEM models can be found in Table S14.

### 3.2 Post Hoc Analyses

Adolescents responding during the pandemic reported significantly more screen time, mental distress, and perceived stress compared to those responding before the pandemic. Girls also reported more conflict with parents, and boys reported exercising more frequently during the pandemic (see Table S15 and Figure S6).

### 3.3 Sensitivity Analyses

Sensitivity analyses excluding participants responding within 2 weeks around the cut‐off date separating pre‐pandemic and pandemic groups revealed similar results as the main analyses (see Supporting Information: Section 8).

## 4 Discussion

In this preregistered cross‐sectional study with data collection over 6 years, we explored levels of eating problems (eating restraint and body concern) and how a range of personal characteristics, including genetic scores for mental health traits were associated with eating problems among 14–16‐year‐old girls and boys before and during the pandemic. We extend previous findings concerning the rise in self‐reported eating problems during the pandemic by separating various eating problem domains in a large, population‐based sample of adolescents of both sexes.

### 4.1 Eating Problems Before and During the Pandemic

In our initial models, we observed more body concern among both boys and girls responding during the pandemic compared to those responding before the pandemic. Girls also reported more eating restraint during the pandemic. The specific eating problems (items) that were more often reported during the pandemic were related to limiting food intake; following dietary rules; fear of losing control over eating; dissatisfaction with the body; feeling uncomfortable with others seeing their own body. In addition to these, girls also reported more often that they allowed thoughts of food, eating, or calories affect concentration; and feeling uncomfortable seeing their own body. These findings are in line with a recent systematic review highlighting a worsening of disordered eating symptoms, including dietary restriction and emotional eating, and a decline in body image outcomes during the Covid‐19 pandemic (Schneider et al. 2023).

A general trend of increasing mental health problems (especially self‐reported), including for eating problems (Galmiche et al. 2019; von Soest and Wichstrøm 2014) has previously been reported. Therefore, the observed differences between those reporting before versus during the pandemic in our models could merely reflect this general ongoing trend. When adjusting for a general linear increase in eating problems over time (i.e., including response year within pre‐pandemic and pandemic times as a covariate), the difference between the pandemic and pre‐pandemic groups of boys was no longer significant. Although our adjustment does not fully cover a 6‐year time‐trend (since it is restricted within the two 3‐year intervals), it appears that general trend of increasing problems might explain the observed differences among boys. Among girls, it appears that the pandemic exposure adds to the general increasing trend, as there are still differences across the pandemic and pre‐pandemic groups also after adjustment.

The mechanisms for increasing eating problems over time/during the pandemic are unclear. However, the result of our post hoc analyses point to factors susceptible to change that could have been intensified during the pandemic. Adolescents responding during the pandemic reported significantly more screen time, and a higher level of mental distress and perceived stress compared to those responding before the pandemic. Girls also reported more conflict with parents. Post hoc analyses indicated that boys exercised more during the pandemic, while girls showed no significant change. The heightened level of exercise among boys may have intensified their focus on health and fitness, potentially exacerbating eating problems.

Our post hoc results did not show increased social media communication during the pandemic. However, it could be that the type of social media used by adolescents changed during the time of data collections. For instance, we know that the use of TikTok increased in Norway between 2020 and 2022 and was used by 73% of Norwegian children (9–18‐year‐olds) in 2022 (Medietilsynet 2022). It is suggested that exposure to idealized body images through social media platforms can exacerbate eating problems (Fioravanti et al. 2022), as adolescents might adopt unhealthy eating patterns and behaviors in an attempt to achieve these unattainable standards.

While we cannot make causal inferences with these post hoc results, it is possible that changes in these factors could be related to increases in eating problems by enhancing pre‐existing vulnerabilities or creating new ones. Future research should investigate the causal relationships between these factors (exercise, screen time, mental distress, stress, and conflict with parents) and increases in eating problems among adolescents.

### 4.2 Associations Between Personal Characteristics and Eating Problems Before and During the Pandemic

As expected, based on much previous research (Fioravanti et al. 2022; Priftis and Panagiotakos 2023; Frieiro Padin et al. 2021; Belcher et al. 2021; Goodwin et al. 2011; Fietz, Touyz, and Hay 2014; Garcia et al. 2020; Henderson et al. 2022; May et al. 2006; Spanos et al. 2010), most of the characteristics included in our models were associated with eating problems. We also expected that some of the associations would change in magnitude when compared across the two groups (pre‐pandemic vs. pandemic). We imagined that when the lives of the adolescents changed so drastically during the first years of the Covid‐19 pandemic, this could also influence factors associated with these problems, and perhaps also introduce some new risk factors. However, for all the variables included—associations remained of the same magnitude across the pre‐pandemic and pandemic groups—suggesting that the pandemic likely intensified existing predictors rather than introducing new ones. However, it is also possible that associations with other factors not measured/included in our models may have changed with the onset of the pandemic. For example, changes in social isolation, sleep patterns, or access to food could have influenced eating behaviors in ways not captured in our study.

There were some interesting sex‐specific differences in the patterns of association between self‐reported characteristics and eating problems. For example, we found that self‐reported screen time was positively associated with body concern among girls, but not boys. There are likely sex differences in the types and content of screen time (e.g., gaming, movies, social media, YouTube). One specific type of screen time—social media communication—was associated with eating restraint in both girls and boys. For girls, time spent communicating on social media was also associated with body concerns. A systematic review recently summarized that social media use was associated with body image concerns, self‐esteem, and eating problems (Frieiro Padin et al. 2021). Future research should investigate how different types of screen time and social media content specifically impact eating behaviors and mental health among adolescents. Understanding these nuances can help develop targeted interventions and guidelines to mitigate possible negative impacts of screen and social media use. Also, being underweight was differently associated with eating problems in boys and girls—associated with less eating restraint among girls and more body concern among boys.

### 4.3 Associations Between Family/Genetic Factors and Eating Problems Before and During the Pandemic

The associations between PGS for mental health traits did also not differ across pre‐pandemic and pandemic groups. PGS for AN was only associated with eating restraint among girls. This may be due to the lower power and predictive accuracy of the AN PGS compared to other mental health PGS, as well as the GWAS includes mainly female cases and controls. Boys may require a greater “genetic dose” of risk genes to develop eating problems (Strober et al. 2001). A possible genetic association in boys could be reflected by the positive association between maternal history of eating disorders and eating restraint in boys.

Across five PGS for related mental health traits, only neuroticism PGS was associated with eating problems. The association with neuroticism PGS is in line with previous observations (Robinson et al. 2020), indicating a possible shared genetic confounding between mental distress/stress and eating problems, as mentioned previously. Genetic liability to higher BMI was associated with both domains of eating problems in girls, following previous research (Robinson et al. 2020; Abdulkadir et al. 2020). For boys, however, BMI PGS was robustly associated with eating restraint but not with shape/weight concern possibly obscuring a U‐shaped association. Notably, low BMI in boys was associated with greater shape/weight concern.

### 4.4 Strengths and Limitations

There are several strengths to this study. First, we have data from a large population‐based sample of girls and boys. As most previous results are based on samples of girls, our results contribute to the existing literature by providing a more detailed understanding of eating problems among boys. Second, we use observational data collected across several years before and during the pandemic allowing us to investigate time trends. Third, our study was preregistered prior to analyses, enhancing the credibility of our findings. However, important limitations should be noted. First, our data on eating problems are cross‐sectional, limiting our ability to draw longitudinal conclusions and infer causal relationships. Nevertheless, our findings are relevant in guiding future research to uncover causality and shared etiology. Second, for some individuals, several eating problem items (e.g., Q1: limiting the amount of food to influence shape/weight) could reflect non‐problematic behaviors related to healthy weight loss or weight management in line with recommended behaviors for overweight adolescents. Third, since we did not find strict measurement invariance across sex, we cannot conclude that the items reflect the same underlying constructs. Following this, it is not meaningful to test the sex differences in the associations observed. Fourth, the psychological and behavioral factors included in our models might have more to do with gender than with sex. However, our data were restricted to sex registered at birth. Fifth, with an increasing trend of eating problems in the young population over the last decades (Galmiche et al. 2019), it is difficult to disentangle this trend from the role of the pandemic. Post‐pandemic measurements will add to this knowledge.

## 5 Conclusion

Our study expands on previous results, showing how the pandemic was associated with increased eating problems among girls but not among boys. The two domains of eating problems, eating restraint and body concern, show somewhat different patterns of correlations with personal characteristics and genetic factors. These associations were similar across pandemic exposure groups. In our data, increasing eating problems during the pandemic were paralleled by increases in screen time, mental distress, stress, exercise, and conflict with parents. These factors could be part of an explanation for increasing eating problems. Our results suggest possible causal factors and gene‐environment interplay in developing eating problems that should be followed up in future studies, and also highlight the importance of recognizing sex‐specific differences in eating problems and tailoring interventions accordingly.

## Author Contributions

**Johanne H. Pettersen:** conceptualization, data curation, formal analysis, investigation, methodology, software, validation, visualization, writing – original draft, writing – review and editing. **Laura Hegemann:** conceptualization, methodology, validation, writing – review and editing. **Kristin Gustavson:** conceptualization, funding acquisition, methodology, project administration, resources, supervision, validation, writing – review and editing. **Ingunn Olea Lund:** validation, writing – review and editing. **Pia Jensen:** validation, writing – review and editing. **Cynthia M. Bulik:** validation, writing – review and editing. **Ole A. Andreassen:** validation, writing – review and editing. **Alexandra Havdahl:** validation, writing – review and editing. **Ragnhild E. Brandlistuen:** conceptualization, funding acquisition, project administration, resources, supervision, validation, writing – review and editing. **Laurie Hannigan:** conceptualization, methodology, validation, writing – review and editing. **Helga Ask:** conceptualization, funding acquisition, investigation, methodology, project administration, resources, supervision, validation, writing – original draft, writing – review and editing.

## Conflicts of Interest

Dr. Andreassen is a consultant to Cortechs.ai and Precision Health and has received speaker's honoraria from Janssen, Otsuka, Sunovion and Lundbeck with no conflict relevant to this work. The other authors declare no conflicts of interest.

## Supporting information


**Data S1.** Supporting Information.

## Data Availability

Data from MoBa used in this study are managed by the national health register holders in Norway (Norwegian Institute of public health) and can be made available to researchers, provided approval from the Regional Committees for Medical and Health Research Ethics (REC), compliance with the EU General Data Protection Regulation (GDPR) and approval from the data owners. The consent given by the participants does not open for storage of data on an individual level in repositories or journals. Researchers who want access to data sets for replication should apply through helsedata.no. Access to data sets requires approval from REC in Norway and an agreement with MoBa.

## References

[eat24314-bib-0001] Abdulkadir, M. , M. Herle , B. L. De Stavola , et al. 2020. “Polygenic Score for Body Mass Index Is Associated With Disordered Eating in a General Population Cohort.” Journal of Clinical Medicine 9, no. 4: 1187. 32326247 10.3390/jcm9041187 PMC7231239

[eat24314-bib-0002] Arnold, P. D. , K. D. Askland , C. Barlassina , et al. 2018. “Revealing the Complex Genetic Architecture of Obsessive‐Compulsive Disorder Using Meta‐Analysis.” Molecular Psychiatry 23, no. 5: 1181–1188. 28761083 10.1038/mp.2017.154 PMC6660151

[eat24314-bib-0003] Belcher, B. R. , J. Zink , A. Azad , C. E. Campbell , S. P. Chakravartti , and M. M. Herting . 2021. “The Roles of Physical Activity, Exercise, and Fitness in Promoting Resilience During Adolescence: Effects on Mental Well‐Being and Brain Development.” Biological Psychiatry: Cognitive Neuroscience and Neuroimaging 6, no. 2: 225–237. 33067166 10.1016/j.bpsc.2020.08.005 PMC7878276

[eat24314-bib-0004] Benjamini, Y. , and Y. Hochberg . 1995. “Controlling the False Discovery Rate: A Practical and Powerful Approach to Multiple Testing.” Journal of the Royal Statistical Society: Series B (Methodological) 57, no. 1: 289–300.

[eat24314-bib-0005] Biele, G. , K. Gustavson , N. O. Czajkowski , et al. 2019. “Bias From Self Selection and Loss to Follow‐Up in Prospective Cohort Studies.” European Journal of Epidemiology 34: 927–938. 31451995 10.1007/s10654-019-00550-1

[eat24314-bib-0006] Biering, K. , N. H. Hjollund , and M. Frydenberg . 2015. “Using Multiple Imputation to Deal With Missing Data and Attrition in Longitudinal Studies With Repeated Measures of Patient‐Reported Outcomes.” Clinical Epidemiology 7: 91–106. 25653557 10.2147/CLEP.S72247 PMC4303367

[eat24314-bib-0007] Carpita, B. , D. Muti , I. M. Cremone , A. Fagiolini , and L. Dell'Osso . 2022. “Eating Disorders and Autism Spectrum: Links and Risks.” CNS Spectrums 27, no. 3: 272–280. 33161925 10.1017/S1092852920002011

[eat24314-bib-0008] Categorical data . n.d. “Categorical Data.” Accessed March 21, 2024. https://lavaan.ugent.be/tutorial/cat.html.

[eat24314-bib-0009] Cerniglia, L. , and S. Cimino . 2022. “Eating Disorders and Internalizing/Externalizing Symptoms in Adolescents Before and During the COVID‐19 Pandemic.” Journal of the American Nutrition Association 42: 445–451. 10.1080/07315724.2022.2063206. 35579970

[eat24314-bib-0010] Cohen, S. , T. Kamarck , and R. Mermelstein . 1983. “A Global Measure of Perceived Stress.” Journal of Health and Social Behavior 24: 385–396. 6668417

[eat24314-bib-0011] Corfield, E. C. , O. Frei , A. A. Shadrin , et al. 2022. The Norwegian Mother, Father, and Child Cohort Study (MoBa) Genotyping Data Resource: MoBaPsychGen Pipeline v.1. BioRxiv, 2022.06.23.496289. 10.1101/2022.06.23.496289.

[eat24314-bib-0012] Costello, A. B. , and J. Osborne . 2019. “Best Practices in Exploratory Factor Analysis: Four Recommendations for Getting the Most From Your Analysis.” Practical Assessment, Research and Evaluation 10, no. 1: 7.

[eat24314-bib-0013] Elkins, I. J. , M. McGue , and W. G. Iacono . 1997. “Genetic and Environmental Influences on Parent–Son Relationships: Evidence for Increasing Genetic Influence During Adolescence.” Developmental Psychology 33, no. 2: 351–363. 9147842 10.1037//0012-1649.33.2.351

[eat24314-bib-0014] Fairburn, C. G. , and S. J. Beglin . 1994. “Assessment of Eating Disorders: Interview or Self‐Report Questionnaire?” International Journal of Eating Disorders 16, no. 4: 363–370. 7866415

[eat24314-bib-0015] Fietz, M. , S. Touyz , and P. Hay . 2014. “A Risk Profile of Compulsive Exercise in Adolescents With an Eating Disorder: A Systematic Review.” Advances in Eating Disorders: Theory, Research and Practice 2, no. 3: 241–263.

[eat24314-bib-0016] Fioravanti, G. , S. Bocci Benucci , G. Ceragioli , and S. Casale . 2022. “How the Exposure to Beauty Ideals on Social Networking Sites Influences Body Image: A Systematic Review of Experimental Studies.” Adolescent Research Review 7, no. 3: 419–458.

[eat24314-bib-0017] Frieiro Padin, P. , R. González Rodríguez , M. D. C. Verde Diego , and P. R. Vázquez . 2021. “Social Media and Eating Disorder Psychopathology: A Systematic Review.” Cyberpsychology Journal of Psychosocial Research on Cyberspace 15, no. 3: Article 6. 10.5817/CP2021-3-6.

[eat24314-bib-0018] Galmiche, M. , P. Déchelotte , G. Lambert , and M. P. Tavolacci . 2019. “Prevalence of Eating Disorders Over the 2000–2018 Period: A Systematic Literature Review.” American Journal of Clinical Nutrition 109, no. 5: 1402–1413. 31051507 10.1093/ajcn/nqy342

[eat24314-bib-0019] Garcia, S. C. , M. E. Mikhail , P. K. Keel , et al. 2020. “Increased Rates of Eating Disorders and Their Symptoms in Women With Major Depressive Disorder and Anxiety Disorders.” International Journal of Eating Disorders 53, no. 11: 1844–1854. 32844425 10.1002/eat.23366 PMC7669595

[eat24314-bib-0020] Goodwin, H. , E. Haycraft , A. M. Willis , and C. Meyer . 2011. “Compulsive Exercise: The Role of Personality, Psychological Morbidity, and Disordered Eating.” International Journal of Eating Disorders 44, no. 7: 655–660. 21321986 10.1002/eat.20902

[eat24314-bib-0021] Gorrell, S. , and S. B. Murray . 2019. “Eating Disorders in Males.” Child and Adolescent Psychiatric Clinics 28, no. 4: 641–651. 10.1016/j.chc.2019.05.012 PMC6785984 31443881

[eat24314-bib-0022] Government.no . n.d. “Timeline: News from Norwegian Ministries about the Coronavirus disease Covid‐19.” Accessed November 14, 2023. https://www.regjeringen.no/en/topics/koronavirus‐covid‐19/timeline‐for‐news‐from‐norwegian‐ministries‐about‐the‐coronavirus‐disease‐covid‐19/id2692402/.

[eat24314-bib-0023] Grove, J. , S. Ripke , T. D. Als , et al. 2019. “Identification of Common Genetic Risk Variants for Autism Spectrum Disorder.” Nature Genetics 51, no. 3: 431–444. 30804558 10.1038/s41588-019-0344-8 PMC6454898

[eat24314-bib-0024] Hammond, R. K. , M. C. Pahl , C. Su , et al. 2021. “Biological Constraints on GWAS SNPs at Suggestive Significance Thresholds Reveal Additional BMI Loci.” eLife 10: e62206. 33459256 10.7554/eLife.62206 PMC7815306

[eat24314-bib-0025] Hawes, M. T. , A. K. Szenczy , D. N. Klein , G. Hajcak , and B. D. Nelson . 2021. “Increases in Depression and Anxiety Symptoms in Adolescents and Young Adults During the COVID‐19 Pandemic.” Psychological Medicine 1–9: 3222–3230. 10.1017/S0033291720005358 PMC7844180 33436120

[eat24314-bib-0026] Henderson, K. A. , N. Obeid , A. Buchholz , et al. 2022. “Coping in Adolescents: A Mediator Between Stress and Disordered Eating.” Eating Behaviors 47: 101626. 36113228 10.1016/j.eatbeh.2022.101626

[eat24314-bib-0027] Hornberger, L. L. , M. A. Lane , M. Lane , et al. 2021. “Identification and Management of Eating Disorders in Children and Adolescents.” Pediatrics 147, no. 1: e2020040279. 33386343 10.1542/peds.2020-040279

[eat24314-bib-0028] Howard, D. M. , M. J. Adams , T.‐K. Clarke , et al. 2019. “Genome‐Wide Meta‐Analysis of Depression Identifies 102 Independent Variants and Highlights the Importance of the Prefrontal Brain Regions.” Nature Neuroscience 22, no. 3: 343–352. 30718901 10.1038/s41593-018-0326-7 PMC6522363

[eat24314-bib-0029] Jorgensen, T. D. , S. Pornprasertmanit , A. M. Schoemann , and Y. Rosseel . 2022. “semTools: Useful Tools for Structural Equation Modeling.”

[eat24314-bib-0030] Lavender, J. M. , T. A. Brown , and S. B. Murray . 2017. “Men, Muscles, and Eating Disorders: An Overview of Traditional and Muscularity‐Oriented Disordered Eating.” Current Psychiatry Reports 19: 1–7. 28470486 10.1007/s11920-017-0787-5 PMC5731454

[eat24314-bib-0031] Lilenfeld, L. R. , W. H. Kaye , C. G. Greeno , et al. 1998. “A Controlled Family Study of Anorexia Nervosa and Bulimia Nervosa: Psychiatric Disorders in First‐Degree Relatives and Effects of Proband Comorbidity.” Archives of General Psychiatry 55, no. 7: 603–610. 9672050 10.1001/archpsyc.55.7.603

[eat24314-bib-0032] Magnus, P. , C. Birke , K. Vejrup , et al. 2016. “Cohort Profile Update: The Norwegian Mother and Child Cohort Study (MoBa).” International Journal of Epidemiology 45, no. 2: 382–388. 10.1093/ije/dyw029. 27063603

[eat24314-bib-0033] Magnus, P. , L. M. Irgens , K. Haug , W. Nystad , R. Skjærven , and C. Stoltenberg . 2006. “Cohort Profile: The Norwegian Mother and Child Cohort Study (MoBa).” International Journal of Epidemiology 35, no. 5: 1146–1150. 10.1093/ije/dyl170. 16926217

[eat24314-bib-0034] May, A. L. , J. Y. Kim , S. M. McHale , and A. C. Crouter . 2006. “Parent–Adolescent Relationships and the Development of Weight Concerns From Early to Late Adolescence.” International Journal of Eating Disorders 39, no. 8: 729–740. 16927386 10.1002/eat.20285

[eat24314-bib-0035] Medietilsynet . 2022. “Barn og unges bruk av sosiale medier.” https://www.medietilsynet.no/globalassets/publikasjoner/barn‐og‐medier‐undersokelser/2022/Barn_og_unges_bruk_av_sosiale_medier.pdf.

[eat24314-bib-0036] Micali, N. , A. E. Field , J. L. Treasure , and D. M. Evans . 2015. “Are Obesity Risk Genes Associated With Binge Eating in Adolescence?” Obesity 23, no. 8: 1729–1736. 26193063 10.1002/oby.21147 PMC4660437

[eat24314-bib-0037] Mora, F. , M. A. Alvarez‐Mon , S. Fernandez‐Rojo , et al. 2022. “Psychosocial Factors in Adolescence and Risk of Development of Eating Disorders.” Nutrients 14, no. 7: 1481. 35406094 10.3390/nu14071481 PMC9002868

[eat24314-bib-0038] Murray, S. B. , S. Griffiths , D. Mitchison , and J. M. Mond . 2017. “The Transition From Thinness‐Oriented to Muscularity‐Oriented Disordered Eating in Adolescent Males: A Clinical Observation.” Journal of Adolescent Health 60, no. 3: 353–355. 10.1016/j.jadohealth.2016.10.014 27989453

[eat24314-bib-0039] Nagel, M. , P. R. Jansen , S. Stringer , et al. 2018. “Meta‐Analysis of Genome‐Wide Association Studies for Neuroticism in 449,484 Individuals Identifies Novel Genetic Loci and Pathways.” Nature Genetics 50, no. 7: 920–927. 29942085 10.1038/s41588-018-0151-7

[eat24314-bib-0040] Neumark‐Sztainer, D. , S. J. Paxton , P. J. Hannan , J. Haines , and M. Story . 2006. “Does Body Satisfaction Matter? Five‐Year Longitudinal Associations Between Body Satisfaction and Health Behaviors in Adolescent Females and Males.” Journal of Adolescent Health 39, no. 2: 244–251. 10.1016/j.jadohealth.2005.12.001 16857537

[eat24314-bib-0041] Nilsen, R. M. , S. E. Vollset , H. K. Gjessing , et al. 2009. “Self‐Selection and Bias in a Large Prospective Pregnancy Cohort in Norway.” Paediatric and Perinatal Epidemiology 23, no. 6: 597–608. 19840297 10.1111/j.1365-3016.2009.01062.x

[eat24314-bib-0042] Norsk Helseinformatikk (NHI) . 2024. “BMI‐kalkulator: Kroppsmasseindeks.” https://nhi.no/skjema‐og‐kalkulatorer/kalkulatorer/diverse/bmi‐kalkulator‐kroppsmasseindeks.

[eat24314-bib-0043] Otto, A. K. , J. M. Jary , J. Sturza , et al. 2021. “Medical Admissions Among Adolescents With Eating Disorders During the COVID‐19 Pandemic.” Pediatrics 148, no. 4: e2021052201. 34244452 10.1542/peds.2021-052201

[eat24314-bib-0044] Paltiel, L. , H. Anita , T. Skjerden , et al. 2014. “The Biobank of the Norwegian Mother and Child Cohort Study–Present Status.” Norsk Epidemiologi 24: 29–35.

[eat24314-bib-0045] Panchal, U. , G. Salazar de Pablo , M. Franco , et al. 2021. “The Impact of COVID‐19 Lockdown on Child and Adolescent Mental Health: Systematic Review.” European Child & Adolescent Psychiatry 32: 1151–1177. 10.1007/s00787-021-01856-w. 34406494 PMC8371430

[eat24314-bib-0046] Pereira, R. F. , and M. Alvarenga . 2007. “Disordered Eating: Identifying, Treating, Preventing, and Differentiating It From Eating Disorders.” Diabetes Spectrum: A Publication of the American Diabetes Association 20, no. 3: 141–148.

[eat24314-bib-0047] Priftis, N. , and D. Panagiotakos . 2023. “Screen Time and Its Health Consequences in Children and Adolescents.” Children 10, no. 10: 1665. 37892328 10.3390/children10101665 PMC10605067

[eat24314-bib-0048] Purves, K. L. , J. R. Coleman , S. M. Meier , et al. 2020. “A Major Role for Common Genetic Variation in Anxiety Disorders.” Molecular Psychiatry 25, no. 12: 3292–3303. 31748690 10.1038/s41380-019-0559-1 PMC7237282

[eat24314-bib-0049] R . 2021. “R: A Language and Environment for Statistical Computing.” R Foundation for Statistical Computing. https://www.R‐project.org/.

[eat24314-bib-0050] Racine, N. , B. A. McArthur , J. E. Cooke , R. Eirich , J. Zhu , and S. Madigan . 2021. “Global Prevalence of Depressive and Anxiety Symptoms in Children and Adolescents During COVID‐19: A Meta‐Analysis.” JAMA Pediatrics 175, no. 11: 1142–1150. 34369987 10.1001/jamapediatrics.2021.2482 PMC8353576

[eat24314-bib-0051] Revelle, W. , and M. W. Revelle . 2015. “Package ‘psych’. The comprehensive R archive network.” Scientific Research 337, no. 338: 161–165.

[eat24314-bib-0052] Robinson, L. , Z. Zhang , T. Jia , et al. 2020. “Association of Genetic and Phenotypic Assessments With Onset of Disordered Eating Behaviors and Comorbid Mental Health Problems Among Adolescents.” JAMA Network Open 3, no. 12: e2026874. 33263759 10.1001/jamanetworkopen.2020.26874 PMC7711322

[eat24314-bib-0053] Rosseel, Y. 2012. “Lavaan: An R Package for Structural Equation Modeling.” Journal of Statistical Software 48: 1–36.

[eat24314-bib-0054] Rubin, D. B. 1987. Multiple Imputation for Survey Nonresponse. New York: Wiley.

[eat24314-bib-0055] Schneider, J. , G. Pegram , B. Gibson , et al. 2023. “A Mixed‐Studies Systematic Review of the Experiences of Body Image, Disordered Eating, and Eating Disorders During the COVID‐19 Pandemic.” International Journal of Eating Disorders 56, no. 1: 26–67. 35322449 10.1002/eat.23706 PMC9087368

[eat24314-bib-0056] Silén, Y. , and A. Keski‐Rahkonen . 2022. “Worldwide Prevalence of DSM‐5 Eating Disorders Among Young People.” Current Opinion in Psychiatry 35, no. 6: 362–371. 36125216 10.1097/YCO.0000000000000818

[eat24314-bib-0057] Smolak, L. , and M. P. Levine . 2015. “Body Image, Disordered Eating, and Eating Disorders: Connections and Disconnects.” In The Wiley handbook of eating disorders, edited by L. Smolak and M. P. Levine , 1–10. Oxford, UK: John Wiley & Sons, Ltd.

[eat24314-bib-0058] Spanos, A. , K. L. Klump , S. A. Burt , M. McGue , and W. G. Iacono . 2010. “A Longitudinal Investigation of the Relationship Between Disordered Eating Attitudes and Behaviors and Parent–Child Conflict: A Monozygotic Twin Differences Design.” Journal of Abnormal Psychology 119, no. 2: 293–299. 20455602 10.1037/a0019028 PMC2941761

[eat24314-bib-0059] Strand, B. H. , O. S. Dalgard , K. Tambs , and M. Rognerud . 2003. “Measuring the Mental Health Status of the Norwegian Population: A Comparison of the Instruments SCL‐25, SCL‐10, SCL‐5 and MHI‐5 (SF‐36).” Nordic Journal of Psychiatry 57, no. 2: 113–118. 12745773 10.1080/08039480310000932

[eat24314-bib-0060] Strober, M. , R. Freeman , C. Lampert , J. Diamond , and W. Kaye . 2000. “Controlled Family Study of Anorexia Nervosa and Bulimia Nervosa: Evidence of Shared Liability and Transmission of Partial Syndromes.” American Journal of Psychiatry 157, no. 3: 393–401. 10698815 10.1176/appi.ajp.157.3.393

[eat24314-bib-0061] Strober, M. , R. Freeman , C. Lampert , J. Diamond , and W. Kaye . 2001. “Males With Anorexia Nervosa: A Controlled Study of Eating Disorders in First‐Degree Relatives.” International Journal of Eating Disorders 29, no. 3: 263–269. 11262504 10.1002/eat.1017

[eat24314-bib-0062] Suarez‐Albor, C. L. , M. Galletta , and E. M. Gómez‐Bustamante . 2022. “Factors Associated With Eating Disorders in Adolescents: A Systematic Review.” Acta Bio Medica: Atenei Parmensis 93, no. 3: e2022253. 10.23750/abm.v93i3.13140 PMC9335430 35775752

[eat24314-bib-0063] Surén, P. , A. B. Skirbekk , L. Torgersen , L. Bang , A. Godøy , and R. K. Hart . 2022. “Eating Disorder Diagnoses in Children and Adolescents in Norway Before vs During the COVID‐19 Pandemic.” JAMA Network Open 5, no. 7: e2222079. 10.1001/jamanetworkopen.2022.22079. 35816316 PMC9280394

[eat24314-bib-0064] Taquet, M. , J. R. Geddes , S. Luciano , and P. J. Harrison . 2022. “Incidence and Outcomes of Eating Disorders During the COVID‐19 Pandemic.” British Journal of Psychiatry 220, no. 5: 262–264. 10.1192/bjp.2021.105 PMC7612698 35048812

[eat24314-bib-0065] Trafford, A. M. , M. J. Carr , D. M. Ashcroft , et al. 2023. “Temporal Trends in Eating Disorder and Self‐Harm Incidence Rates Among Adolescents and Young Adults in the UK in the 2 Years Since Onset of the COVID‐19 Pandemic: A Population‐Based Study.” Lancet Child & Adolescent Health 7, no. 8: 544–554. 37352883 10.1016/S2352-4642(23)00126-8

[eat24314-bib-0066] Uhlhaas, P. J. , C. G. Davey , U. M. Mehta , et al. 2023. “Towards a Youth Mental Health Paradigm: A Perspective and Roadmap.” Molecular Psychiatry 28: 3171–3181. 37580524 10.1038/s41380-023-02202-z PMC10618105

[eat24314-bib-0067] Unnarsdóttir, A. B. , A. Lovik , C. Fawns‐Ritchie , et al. 2022. “Cohort Profile: COVIDMENT: COVID‐19 Cohorts on Mental Health Across Six Nations.” International Journal of Epidemiology 51, no. 3: e108–e122. 35020900 10.1093/ije/dyab234 PMC8690101

[eat24314-bib-0068] Varela, C. , Á. Hoyo , M. E. Tapia‐Sanz , et al. 2023. “An Update on the Underlying Risk Factors of Eating Disorders Onset During Adolescence: A Systematic Review.” Frontiers in Psychology 14: 14. 10.3389/fpsyg.2023.1221679 PMC10663237 38023032

[eat24314-bib-0069] Verbeke, M. , D. Schrans , S. Deroose , and J. De Maeseneer . 2006. “The International Classification of Primary Care (ICPC‐2): An Essential Tool in the EPR of the GP.” Studies in Health Technology and Informatics 124: 809–814. 17108613

[eat24314-bib-0070] von Soest, T. , and L. Wichstrøm . 2014. “Secular Trends in Eating Problems Among Norwegian Adolescents From 1992 to 2010.” International Journal of Eating Disorders 47, no. 5: 448–457. 24610169 10.1002/eat.22271

[eat24314-bib-0071] Watson, H. J. , Z. Yilmaz , L. M. Thornton , et al. 2019. “Genome‐Wide Association Study Identifies Eight Risk Loci and Implicates Metabo‐Psychiatric Origins for Anorexia Nervosa.” Nature Genetics 51, no. 8: 1207–1214. 10.1038/s41588-019-0439-2. 31308545 PMC6779477

[eat24314-bib-0072] World Health Organization . 1993. The ICD‐10 Classification of Mental and Behavioural Disorders: Diagnostic Criteria for Research. Vol. 2. Geneva, Switzerland: World Health Organization.

[eat24314-bib-0073] Yilmaz, Z. , J. A. Hardaway , and C. M. Bulik . 2015. “Genetics and Epigenetics of Eating Disorders.” Advances in Genomics and Genetics 5: 131–150. 27013903 10.2147/AGG.S55776 PMC4803116

[eat24314-bib-0074] Yilmaz, Z. , K. Schaumberg , M. Halvorsen , et al. 2022. “Predicting Eating Disorder and Anxiety Symptoms Using Disorder‐Specific and Transdiagnostic Polygenic Scores for Anorexia Nervosa and Obsessive‐Compulsive Disorder.” Psychological Medicine 1–15: 3021–3035. 10.1017/S0033291721005079 PMC9440960 35243971

