# Supplementary material for: Eating Problems Among Adolescent Boys and Girls Before and During the Covid‐19 Pandemic
Source: Int J Eat Disord. 2024 Oct 30;58(1):193–205. doi: 10.1002/eat.24314 (PMC11784851; doi:10.1002/eat.24314)
Supplement: Supplementary file 1 — Data S1. Supporting Information. [file EAT-58-193-s001.docx]

**Supporting Information**

**Eating problems among adolescent boys and girls before and during the Covid-19 pandemic**

Table of Contents

[**Table S1: Deviations from the preregistered analysis plan, following the Preregistration Deviation Table Template^1^** 4](#_Toc180943089)

[**Figure S1: Flow chart of study participants** 6](#_Toc180943090)

[**Section 1: Eating problem items** 7](#_Toc180943091)

[**Section 2: Exploratory factor analysis of eating problem items** 7](#_Toc180943092)

[**Table S2: Fit measures EFA of nine eating problem items (n= 22706, boys and girls)** 7](#_Toc180943093)

[**Table S3: EFA factor loadings from the 2-factor model (boys and girls)** 7](#_Toc180943094)

[**Section 3: Confirmatory factor analysis of eating problems** 8](#_Toc180943095)

[**Table S4: CFA comparison (boys and girls)** 8](#_Toc180943096)

[**Section 4: Measurement invariance testing across sex** 8](#_Toc180943097)

[**Table S5: Measurement invariance testing across sex** 9](#_Toc180943098)

[**Section 5: Preparing/defining eating problem outcomes** 9](#_Toc180943099)

[**Figure S2: Factor structure for eating problems in girls and boys** 10](#_Toc180943100)

[**Table S6: Fit measures EFA (boys and girls separately)** 11](#_Toc180943101)

[**Table S7: EFA loadings (2 factor model) (girls and boys separately)** 11](#_Toc180943102)

[**Table S8: CFA fit comparison (girls and boys separately)** 12](#_Toc180943103)

[**Section 6: Preparing/defining covariates** 13](#_Toc180943104)

[**Section 7: Measurement invariance testing across pandemic groups and latent mean comparisons** 14](#_Toc180943105)

[**Table S9: Measurement invariance across pandemic groups** 15](#_Toc180943106)

[**Table S10: Chi-square trend test for each item across pandemic exposure for girls and boys** 16](#_Toc180943107)

[**Figure S3: Mean score for each eating problem item across sex and year of responding to questionnaire** 17](#_Toc180943108)

[**Figure S4: Standardized estimates from SEM models of associations between predictors and eating problem domains** 18](#_Toc180943109)

[**Figure S5: Standardized estimates from SEM models of associations between PGS and eating problem domains** 19](#_Toc180943110)

[**Table S11: Standardized estimates from SEM models for girls and boys** 20](#_Toc180943111)

[**Table S12: Model fit comparison between pre-pandemic and pandemic groups across free and constrained parameters among girls** 22](#_Toc180943112)

[**Table S13: Model fit comparison between pre-pandemic and pandemic groups across free and constrained parameters among boys** 24](#_Toc180943113)

[**Table S14: Model fit indices for SEM and multigroup SEM models** 26](#_Toc180943114)

[**Figure S6: Mean score for each predictor across sex and year of responding to questionnaire** 27](#_Toc180943115)

[**Table S15: Mean differences in covariates among adolescents before and during the pandemic** 28](#_Toc180943116)

[**Section 8: Sensitivity analyses** 29](#_Toc180943117)

[**Supplementary references** 30](#_Toc180943118)

## **Table S1: Deviations from the preregistered analysis plan, following the Preregistration Deviation Table Template^1^**

| **Deviations** | | | | | |
| --- | --- | --- | --- | --- | --- |
| **#** | **Details** | | **Original wording** | **Deviation Description** | **Reader Impact** |
| 1 | Type | Analysis | To investigate the first hypothesis (H1) we will run a group CFA across pandemic exposure. The null hypothesis will be supported/rejected based on the p value from the likelihood ratio test comparing means of latent variables constrained vs freed across exposure groups. | As we did measurement invariance testing and strong invariance was achieved between pandemic groups, we could compare factor mean differences across pandemic groups by constraining means of be 0 in the pre-pandemic group and then evaluate mean difference of the two latent factors in a multi group SEM model with year answered as a covariate to adjust for time. The null hypothesis was supported/rejected based on the p value of the latent mean difference. | This change was made to reduce number of analyses as the strong invariance which enables mean comparison across group was achieved. This should have little influence over final results, but the change allowed us to test for significance within each factor and adjust for possible time effects |
|  | Reason | Other (Please Explain) |  |  |  |
|  | Timing | After results known |  |  |  |
| 2 | Type | Analysis | For the SEM models we will use full information maximum likelihood (FIML) estimation to handle missing data. | FIML is currently not an available option for categorical data using the WLSMV optimizer in lavaan. The alternative option was pairwise deletion which only keeps complete data. Therefore, multiple imputation was used to handle missing data. | This deviation allows us to use more available data compared to pairwise deletion, which can lead to more statistically powered and less biased estimates. |
|  | Reason | Typo/Error |  |  |  |
|  | Timing | After data access |  |  |  |
| 3 | Type | Analysis | Measurement invariance across pandemic groups was specified as exploratory analyses. | Measurement invariance testing between pandemic groups was moved to the first step of the main analyses as we realized the necessity of establishing measurement invariance to be able to compare mean differences across groups. | This change should have no influence over results or interpretation of results. |
|  | Reason | Other (Please Explain) |  |  |  |
|  | Timing | After data access |  |  |  |
| 4 | Type | Variables | Year born was specified as a covariate. | To adjust for time we instead selected year answered as a covariate to more correctly adjust for time effects as some adolescents from different cohorts answered within the same years. | This change allows us to adjust for effects over time, as there is an already increasing trend of eating problems. |
|  | Reason | Typo/Error |  |  |  |
|  | Timing | After data access |  |  |  |
| 5 | Type | Sample | In the preregistration we specified the sample to be 21,957. | After gaining access to updated questionnaire data the sample increased to 22,706. | This change should have little influence on the results. |
|  | Reason | New knowledge |  |  |  |
|  | Timing | After data access |  |  |  |
| 6 | Type | Variables | In the preregistration, the cut-off data separating the pre-pandemic group from the pandemic group was set to be March 12^th^. | This was an error, and the cut-off date was changed to April 12^th^ to account for participants being asked about the last 4 weeks. | This change should have no influence on the results. |
|  | Reason | Typo/Error |  |  |  |
|  | Timing | After data access |  |  |  |
| 7 | Type | Variables | Eating problem item 9 was specified to have 5 responses. | This item was changed to 3 responses by combining the extreme ends so that the variable would go in one direction since we believed that girls and boys might be affected by either end of the scale. The question originally has the options: too thin/a little too thin/okay/a little too thick/too thick. The responses were changed to: okay/a little too thin/thick/too thin/thick. | This deviance allowed us to investigate both ends of this spectrum as being problematic. |
|  | Reason | Typo/Error |  |  |  |
|  | Timing | After data access |  |  |  |
| **Unregistered steps** | | | | | |
| **#** | **Details** | | **Original wording** | **Deviation Description** | **Reader Impact** |
| 1 | Type | Variables | We did not specify including variables on BMI in the preregistration. | As BMI is very relevant to the topic of the paper, we chose to include BMI and polygenic risk for BMI as predictors in the analyses. | Including BMI and its polygenic score as variables enriches the research by investigating and adjusting for BMI. This addition might contribute to the understanding of eating problems. |
|  | Reason | New knowledge |  |  |  |
|  | Timing | After data access |  |  |  |
| 2 | Type | Variable | We did not specify including polygenic score of autism in the preregistration. | After a more thorough literature search and feedback from collaborators, we thought it would be important to add autism in the analyses as some of the items asked could relate to traits common to individuals with autism. | This deviation adds to the depth to the analysis by considering a genetic component that may influence the traits under study. Readers will gain a more nuanced understanding of how genetic liability to autism might influence eating problems. |
|  | Reason | New knowledge |  |  |  |
|  | Timing | After data access |  |  |  |

## **Figure S1: Flow chart of study participants**


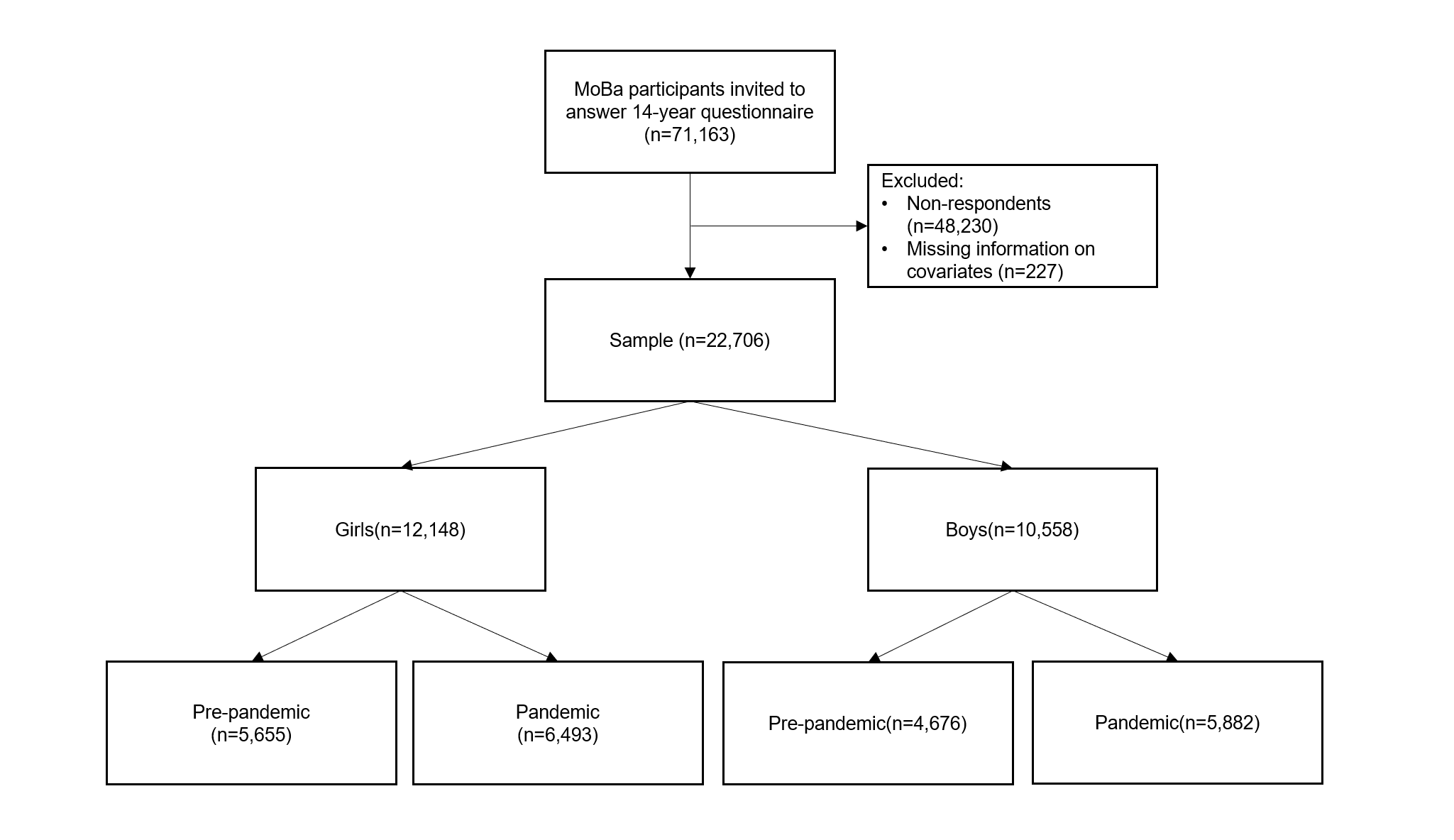


**Note:** Pre-pandemic was here defined as those answering before the cut-off date (April 12^th^, 2020), and pandemic was defined as those answering after this cut-off date (until December 2022).

## **Section 1: Eating problem items**

The 8 items of the EDE-Q were: *“Over the past 4 weeks, how often 1) have you been deliberately trying to limit the amount of food you eat to influence your shape or weight? 2) have you tried to follow definite rules regarding what you can eat, in order to influence your shape or weight? 3) have you had a definite fear of losing control over eating? 4) has thinking about food, eating or calories made it very difficult to concentrate on things you are interested in? 5) have you eaten secretly? 6)* *how dissatisfied have you been with your shape? 7) how uncomfortable have you felt seeing your own body? 8) how uncomfortable have you felt about others seeing your shape or figure?* The last item was: “*How do you consider your own weight?”.*

## **Section 2: Exploratory factor analysis of eating problem items**

Parallel analyses suggested 3 underlying factors. Three separate EFAs were therefore run with 1, 2 and 3 factors indicating better fit with increasing number of factors (see Table S2). EFA with 2 and 3 factors showed acceptable fit with CFI above 0.95, RMSEA below 0.08 and SRMR below 0.06).^2^ However, inspecting the structure including 3 factors revealed that only two items loaded highest one of the factors which goes against recommendations for having at least three items per factor^3^. The EFA with a two-factor structure was therefore selected. See Table S3, for factor loadings.

## **Table S2: Fit measures EFA of nine eating problem items (n= 22706, boys and girls)**

| **Model** | **Chi-squared scaled** | **df** | **P value scaled** | **CFI scaled** | **RMSEA scaled** | **SRMR** |
| --- | --- | --- | --- | --- | --- | --- |
| 1 factor | 4751 | 27 | **<0.001** | 0.965 | 0.120 | 0.062 |
| 2 factors | 512 | 19 | **<0.001** | 0.996 | 0.049 | 0.021 |
| 3 factors | 178 | 12 | **<0.001** | 0.999 | 0.036 | 0.010 |

**Note**: Fit indices suggest the three-factor model provides the best fit to the data, as indicated by the highest CFI value and lowest RMSEA and SRMR values, which fall below the recommended thresholds for a good fit. df = degrees of freedom.

## **Table S3: EFA factor loadings from the 2-factor model (boys and girls)**

| **Items** | **Factor 1** | **Factor 2** | **H2** | **U2** | **Com** |
| --- | --- | --- | --- | --- | --- |
| Q1 | 0.07 | 0.84 | 0.81 | 0.19 | 1.0 |
| Q2 | -0.11 | 0.94 | 0.74 | 0.26 | 1.0 |
| Q3 | 0.24 | 0.66 | 0.72 | 0.28 | 1.3 |
| Q4 | 0.47 | 0.45 | 0.73 | 0.27 | 2.0 |
| Q5 | 0.22 | 0.26 | 0.20 | 0.80 | 1.9 |
| Q6 | 0.83 | 0.09 | 0.81 | 0.19 | 1.0 |
| Q7 | 0.94 | 0.00 | 0.88 | 0.12 | 1.0 |
| Q8 | 0.89 | -0.07 | 0.71 | 0.29 | 1.0 |
| Q9 | 0.60 | 0.05 | 0.41 | 0.59 | 1.0 |

**Note**: The table outlines the factor loadings on Factor 1 and Factor 2 for nine items, the communality (H2), the uniqueness (U2) and the complexity (Com) of the items. Factor loadings represent the correlation of each item with the underlying factor, where higher values indicate a stronger association. Communality values indicate the proportion of variance in each item explained by the two factors, while uniqueness represents the item-specific variance not explained by the factors. Complexity values denote the number of factors each item significantly loads on, with a value of 1.0 indicating loading on a single factor.

## **Section 3: Confirmatory factor analysis of eating problems**

One of the eating problem items (Q4: Thought of food, eating or calories affecting concentration) loaded similarly on the two underlying factors in the best fitting EFA (i.e., 0.47 and 0.45). Two separate CFA were run with Q4 loading on one factor in the first model and on the other factor in the second. Model comparison revealed better fit for the first model showing preference for items Q1-Q5 on the latent factor 1 and Q6-Q9 on the latent factor 2 (Table S4). The CFA model exhibited a good fit with CFI above the acceptable threshold of 0.95, RMSEA below the 0.08 threshold and SRMR below 0.06.

## **Table S4: CFA comparison (boys and girls)**

| **Model** | **Model equations** | **Chi-squared scaled** | **df** | **P value scaled** | **CFI scaled** | **RMSEA scaled** | **SRMR** |
| --- | --- | --- | --- | --- | --- | --- | --- |
| 1 | F1=~Q1+Q2+Q3+Q4+Q5  F2=~Q6+Q7+Q8+Q9 | 1662 | 26 | **<0.001** | 0.986 | 0.076 | 0.037 |
| 2 | F1=~Q1+Q2+Q3+Q5  F2=~Q4+Q6+Q7+Q8+Q9 | 2204 | 26 | **<0.001** | 0.982 | 0.087 | 0.044 |

**Note**: Two CFA models are compared (one with Q4 loading on factor 1 and the other model with Q4 loading on F2). Fit indices indicates the best fit for model 1, with highest CFI value and lowest RMSEA and SRMR. CFI: Comparative fit index; RMSEA: Root mean square error of approximation; SRMR: Standardized root mean square residual.

## **Section 4: Measurement invariance testing across sex**

First, we examined the fit of the baseline model in each group separately. We then tested for threshold invariance to consider if the response scales of the items were used similarly by boys and girls. The third step was to test for strong invariance to see if the underlying factors were assumed to be the same constructs across sex. Lastly, we tested for strict invariance, setting the residual variance to be constant across sex. Models were run using the WLSMV estimator in the lavaan and semTools package^4^. To compare model fit, we selected the Comparative fit index (CFI) with a criterion of -Δ0.01, Root mean square error of approximation (RMSEA) with a criterion of Δ0.015 and the Standardized root mean square residual (SRMR) with the criteria of Δ0.03 for threshold invariance, and Δ0.01 for strong and strict invariance^5^. If the measurement invariance testing does not hold at each step, we will run the following analyses separately for girls and boys.

Measurement invariance testing revealed measurement differences. Table S5 shows model fit comparison across measurement invariance steps. At the configural step, the baseline model showed excellent model fit for both groups (CFI = 0.995, RMSEA = 0.027, TLI = 0.993, SRMR = 0.032). Next, factor loadings were constrained to be equal across groups resulting in a slight decrease in CFA (ΔCFI = -0.003), a small increase in RMSEA (ΔRMSEA = 0004) and a small increase in SRMR (ΔSRMR = 0.006) which did not exceed the commonly used thresholds (ΔCFI < -0.01, ΔRMSEA <0.015, ΔSRMR <0.03), indicating that threshold invariance held at this step (CFI = 0.992, RMSEA = 0.031, TLI = 0.990, SRMR = 0.038). At the strong invariance step, factor loadings and intercepts were constrained across sex. There was a small reduction in in CFI (ΔCFI = -0.01), an acceptable increase in RMSEA (ΔRMSEA = 0.013) and a small increase in SRMR (ΔSRMR = 0.009) suggesting that strong invariance was supported at this step (CFI = 0.982, RMSEA = 0.044, TLI = 0.981, SRMR =0.047). At the final step residual variances were constrained to be equal across sex resulting in a slight decrease in CFI (ΔCFI = -0.006), a small increase in RMSEA (ΔRMSEA= 0.004), but a greater in increase in SRMR (ΔSRMR = 0.018) resulting in a decreased model fit (ΔCFI = 0.976, RMSEA = 0.048, TLI = 0.977, SRMR = 0.065). The changes in CFI and RMSEA remained within the accepted range, but the increase in SRMR suggests possible issues with strict invariance.

## **Table S5: Measurement invariance testing across sex**

| **Model** | **Compared to** | **CFI** | **ΔCFI** | **RMSEA** | **ΔRMSEA** | **TLI** | **SRMR** | **ΔSRMR** | **Invariance holds** |
| --- | --- | --- | --- | --- | --- | --- | --- | --- | --- |
| Configural |  | 0.995 |  | 0.027 |  | 0.993 | 0.032 |  |  |
| Threshold | Configural | 0.992 | -0.003 | 0.031 | 0.004 | 0.990 | 0.038 | 0.006 | Yes |
| Strong | Threshold | 0.982 | -0.01 | 0.044 | 0.013 | 0.981 | 0.047 | 0.009 | Yes |
| Strict | Strong | 0.976 | -0.006 | 0.048 | 0.004 | 0.977 | 0.065 | 0.018 | Yes/No |

**Note:** The models tested for measurement invariance were configural, threshold, strong and strict invariance. Invariance held at the threshold and strong step, but only partially for strict invariance. CFI: Comparative fit index; RMSEA: Root mean square error of approximation; SRMR: Standardized root mean square residual; TLI: Tucker-Lewis index.

## **Section 5: Preparing/defining eating problem outcomes**

*Sex-stratified EFA*

Following evidence from measurement invariance testing across sex, separate EFA were run for girls and boys. A random half of the sex-stratified samples were selected for EFA analysis for girls (N = 6,074) and boys (N=5279). Parallel analyses suggested 3 factors and 1 component for both girls and boys. Three separate EFA were therefore run with 1, 2 and 3 factors indicating better fit with increasing number of factors (see Table S6). However, similarly to the EFA from the total sample, only 2 items loaded highest on the third factor (ML2 for girls, and ML3 for boys). The two-factor structure was therefore selected for both. See Table S7, for EFA loadings.

*Sex-stratified CFA*

CFA were run on the remaining random half of each sample for girls (N = 6074) and boys (N = 5279). For girls and boys, the factor loadings from EFA revealed similar loadings on the two factors for Q4 and Q5. Three separate CFA were therefore run to compare model fit. For both girls and boys, the first model with Q1:Q5 loading on one factor and Q6:Q9 loading on the other showed the best model fit (Table S8). For girls, the CFI was above the acceptable threshold of 0.95, RMSEA was right above the 0.08 threshold indicating a marginal fit, and SRMR was below 0.06. For boys, CFA, RMSEA and SRMR indicated a good model fit.

## **Figure S2: Factor structure for eating problems in girls and boys**


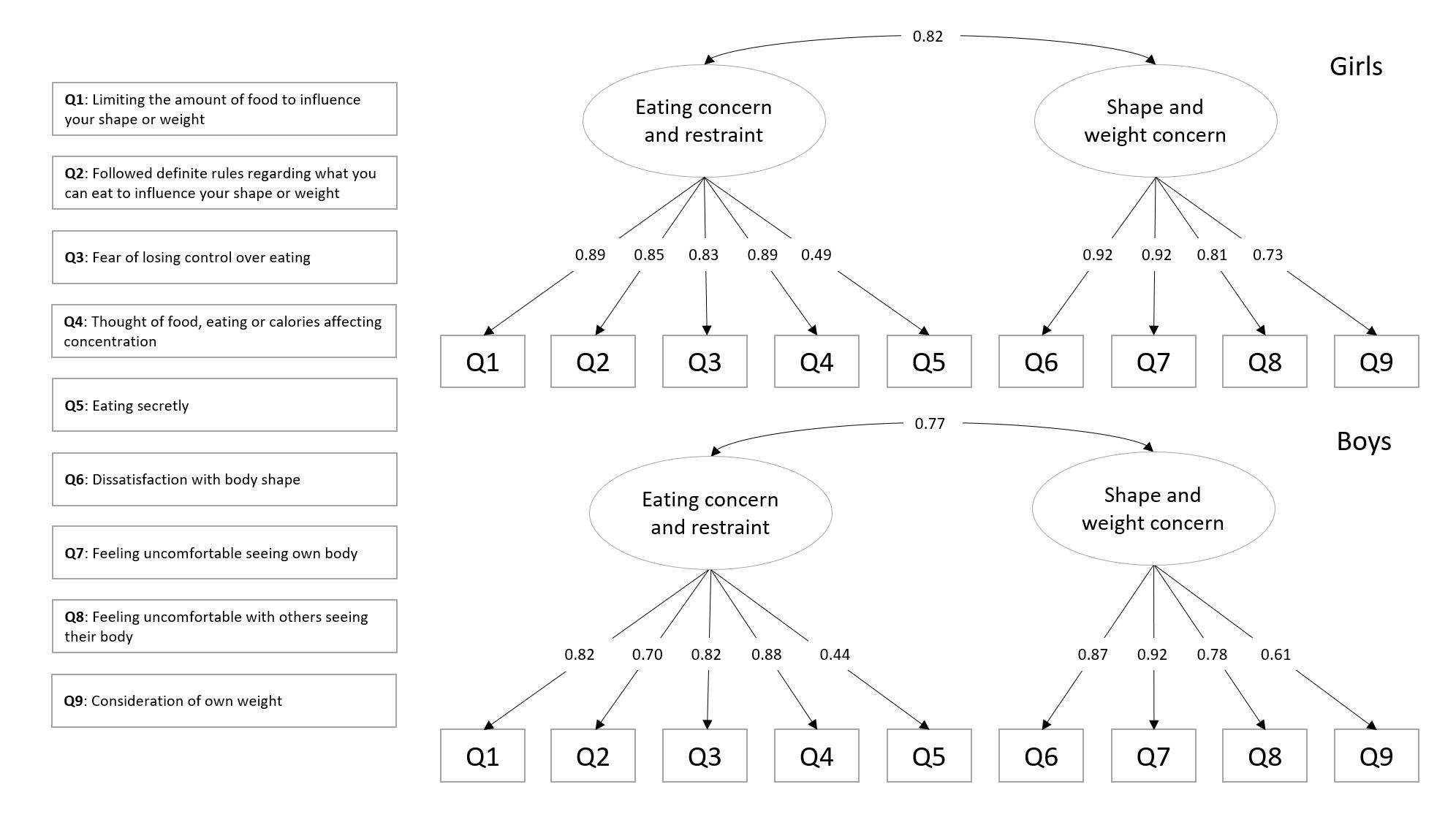


**Note:** Factor structure with factor loadings for the two latent factors (Eating concern and restraint and Shape and weight concern) for girls and boys. The column on the left displays the 9 eating problem items (Q1-Q9).

## **Table S6: Fit measures EFA (boys and girls separately)**

|  | **Factors** | **Chi-squared scaled** | **df** | **P value scaled** | **CFI scaled** | **RMSEA scaled** | **SRMR** |
| --- | --- | --- | --- | --- | --- | --- | --- |
| **Girls** |  |  |  |  |  |  |  |
|  | 1 | 2947 | 27 | **<0.001** | 0.963 | 0.136 | 0.067 |
|  | 2 | 315 | 19 | **<0.001** | 0.996 | 0.052 | 0.019 |
|  | 3 | 103 | 12 | **<0.001** | 0.999 | 0.036 | 0.010 |
| **Boys** |  |  |  |  |  |  |  |
|  | 1 | 1483 | 27 | **<0.001** | 0.938 | 0.103 | 0.079 |
|  | 2 | 334 | 19 | **<0.001** | 0.987 | 0.057 | 0.035 |
|  | 3 | 92 | 12 | **<0.001** | 0.997 | 0.036 | 0.015 |

**Note**: Fit indices suggest the three-factor model provides the best fit to the data for girls and boys, as indicated by the highest CFI value and lowest RMSEA and SRMR values, which fall below the recommended thresholds for a good fit. df = degrees of freedom. CFI: Comparative fit index; RMSEA: Root mean square error of approximation; SRMR: Standardized root mean square residual.

## **Table S7: EFA loadings (2 factor model) (girls and boys separately)**

|  | **Items** | **Factor 1** | **Factor 2** | **H2** | **U2** | **Com** |
| --- | --- | --- | --- | --- | --- | --- |
| **Girls** |  |  |  |  |  |  |
|  | Q1 | 0.04 | 0.88 | 0.83 | 0.17 | 1.0 |
|  | Q2 | -0.08 | 0.96 | 0.81 | 0.19 | 1.0 |
|  | Q3 | 0.16 | 0.71 | 0.69 | 0.31 | 1.1 |
|  | Q4 | 0.41 | 0.49 | 0.71 | 0.29 | 2.0 |
|  | Q5 | 0.22 | 0.31 | 0.24 | 0.76 | 1.8 |
|  | Q6 | 0.81 | 0.13 | 0.83 | 0.17 | 1.1 |
|  | Q7 | 0.93 | -0.01 | 0.86 | 0.14 | 1.0 |
|  | Q8 | 0.90 | -0.09 | 0.70 | 0.30 | 1.0 |
|  | Q9 | 0.62 | 0.12 | 0.51 | 0.49 | 1.1 |
| **Boys** |  |  |  |  |  |  |
|  | Q1 | 0.07 | 0.80 | 0.72 | 0.28 | 1.0 |
|  | Q2 | -0.10 | 0.88 | 0.66 | 0.34 | 1.0 |
|  | Q3 | 0.31 | 0.57 | 0.64 | 0.36 | 1.6 |
|  | Q4 | 0.49 | 0.40 | 0.65 | 0.35 | 1.9 |
|  | Q5 | 0.22 | 0.23 | 0.17 | 0.83 | 2.0 |
|  | Q6 | 0.79 | 0.09 | 0.72 | 0.28 | 1.0 |
|  | Q7 | 0.90 | 0.01 | 0.83 | 0.17 | 1.0 |
|  | Q8 | 0.84 | -0.06 | 0.65 | 0.35 | 1.0 |
|  | Q9 | 0.66 | -0.08 | 0.37 | 0.63 | 1.0 |

**Note**: The table outlines the factor loadings on Factor 1 and Factor 2 for nine items, the communality (H2), the uniqueness (U2) and the complexity (Com) of the items for boys and girls.

## **Table S8: CFA fit comparison (girls and boys separately)**

|  | **Model** | **Model equation** | **Chi-squared scaled** | **df** | **P value scaled** | **CFI scaled** | **RMSEA scaled** | **SRMR** |
| --- | --- | --- | --- | --- | --- | --- | --- | --- |
| Girls |  |  |  |  |  |  |  |  |
|  | 1 | F1=~Q1+Q2+Q3+Q4+Q5  F2=~Q6+Q7+Q8+Q9 | 1123 | 26 | **<0.001** | 0.985 | 0.085 | 0.041 |
|  | 2 | F1=~Q1+Q2+Q3+Q5  F2=~Q4+Q6+Q7+Q8+Q9 | 1446 | 26 | **<0.001** | 0.981 | 0.097 | 0.047 |
|  | 3 | F1=~Q1+Q2+Q3+Q4  F2=~Q5+Q6+Q7+Q8+Q9 | 1239 | 26 | **<0.001** | 0.984 | 0.084 | 0.045 |
| Boys |  |  |  |  |  |  |  |  |
|  | 1 | F1=~Q1+Q2+Q3+Q4+Q5  F2=~Q6+Q7+Q8+Q9 | 556 | 26 | **<0.001** | 0.977 | 0.063 | 0.046 |
|  | 2 | F1=~Q1+Q2+Q3+Q5  F2=~Q4+Q6+Q7+Q8+Q9 | 638 | 26 | **<0.001** | 0.974 | 0.068 | 0.053 |
|  | 3 | F1=~Q1+Q2+Q3+Q4  F2=~Q5+Q6+Q7+Q8+Q9 | 665 | 26 | **<0.001** | 0.973 | 0.069 | 0.055 |

**Note**: Three CFA models are compared (with Q4 and Q5 loading on each factor combined and separately). Fit indices suggest that the best fit was model 1 for girls and boys, with highest CFI value and lowest RMSEA and SRMR. CFI: Comparative fit index; RMSEA: Root mean square error of approximation; SRMR: Standardized root mean square residual.

## **Section 6: Preparing/defining covariates**

*Exercise*

The 14-year questionnaire included two versions of questions about exercise. In the first version of the questionnaire (8% of the participants) the adolescents were asked: *Outside of school hours, about how many hours a week do you usually do sports or physical activities (soccer, handball, running, gymnastics, dance etc.) so much that you become out of breath or sweaty? (Never/Less than 1 hour/1-2 hours/3-4 hours/5-7 hours/8-10 hours/11 hours or more).* In the second version of the questionnaire, the adolescents were asked: *Outside school hours, about how many hours a week are you physically active? (Less than 1 hour/1-2 hours/3-4 hours/5-7 hours/8-10 hours/11 hours or more).*

*Social media communication*

One item was used to measure social media communication: *How much time do you usually spend during one weekday on communicating with friends on social media* (Never or rarely/Less than 1 hour/1-2 hours/3-4 hours/5-6 hours/7 hours or more).

*Screen time*

Similarly, a variable was created for screen time: *How much time do you usually spend during one weekday on sitting/lying down with PC, mobile or tablet* (Never or rarely/Less than 1 hour/1-2 hours/3-4 hours/5-6 hours/7 hours or more). Both variables were scored from 1 (Never) to 6 (7 hours or more).

*Body Mass Index*

Using the “fastDummies” R package, we created two dummy variables with the “Normal weight” category set as reference. The two dummy variables were: “Underweight vs. Normal weight” and “Overweight vs. Normal weight”. Body Mass Index was not responded to in 2017.

*Hopkins Symptom Checklist (SCL-10)*

The SCL-10 is a shortened version of the complete 90-item SCL-90, designed for screening of anxiety and depression symptoms with 5 items each. Cronbach’s alpha of SCL-10 was 0.92. SCL-10 was not responded to in 2017.

*Perceived Stress Scale (PSS-4)*

The Cronbach’s alpha of PSS-4 was 0.63.

*Parent-child conflict*

The adolescents were asked: *My parents criticize me; my parents irritate me; my parents hurt my feelings and my parents and I get into arguments (1-Never/2-Now and then/3-Often/4-Almost all the time).* The Cronbach’s alpha for this scale was 0.72.

*Mothers’ history of eating disorder*

The “Phenotools” R package^6^ was used to code individuals with “Yes” if they were ever diagnosed in NPR with the ICD-10 code for eating disorders (“F50”) or ever registered in KURH with the ICPC-2 code for Anorexia nervosa/bulimia (“P86”). Additionally, the mothers answered an item in the MoBa questionnaires sent out when they were 6 months pregnant asking whether they ever had received a diagnosis for eating disorders.

*Polygenic scores*

PRSice2^7^ was used to create polygenic scores (PGS) for each adolescent based on summary statistics from genome-wide association studies (GWAS). The summary statistics for each PGS were filtered for minor allele frequencies (MAF>1%) and INFO threshold >.8. A range of p-value thresholds was selected to create the PGS (<5e-08, <1e-6, <1e-4, <.001, <.01, <.05, <.1, <.2, <.5 and 1). Further, we used principal component analysis (PCA) on these 10 PGS (calculated based on different thresholds) and used the first PGS principal component as the predictor variable in our models^8^. This approach will reduce the risk of type 1 error (false positives) and overfitting.

*Year of response*

Year of response was coded from 0-2 within the pre-pandemic and pandemic period (2017=0, 2018=1, 2019=2, 2020=0, 2021=1, 2022=2)

## **Section 7: Measurement invariance testing across pandemic groups and latent mean comparisons**

Measurement invariance testing across pandemic groups was conducted using multi-group CFA as an exploratory analysis for girls and boys separately using similar steps as previously (see Table S9). At the configural step, the baseline models showed excellent fit for both groups (girls: CFI = 0.096, RMSEA = 0.027, TLI = 0.995, SRMR = 0.029, boys: CFI = 0.991, RMSEA = 0.023, TLI = 0.987, SRMR = 0.036). Next, factor loadings were constrained to be equal across groups resulting in a stable CFA for girls and a slight decrease in CFA for boys (girls: ΔCFI = 0, boys: ΔCFI = -0.001) and a stable RMSEA for boys and girls (girls: ΔRMSEA = 0, boys: ΔRMSEA = 0). As the change in CFA was less than the commonly used threshold (ΔCFI < 0.01), threshold invariance held at this step (girls: CFI = 0.996, RMSEA = 0.027, TLI = 0.995, SRMR = 0.031, boys: CFI = 0.990, RMSEA = 0.023, TLI = 0.988, SRMR = 0.037). At the strong invariance step, factor loadings and intercepts were constrained across pandemic exposure. There was a small reduction in in CFI (girls: ΔCFI = -0.001, boys: ΔCFI = -0.001) and a small increase in RMSEA for girls (girls: ΔRMSEA = 0.002, boys: ΔRMSEA = 0) suggesting that strong invariance was supported at this step (girls: CFI = 0.995, RMSEA = 0.029, TLI = 0.995, SRMR =0.033, boys: CFI = 0.988, RMSEA = 0.023, TLI = 0.987, SRMR =0.038). At the final step residual variances were constrained to be equal across pandemic exposure resulting in a small decrease in CFA among boys (girls: ΔCFI = 0, boys: ΔCFI = -0.001) and a small decrease in RMSEA for girls (girls: ΔRMSEA = -0.001, boys: ΔRMSEA = 0). This indicated invariance at the final step (girls: CFI = 0.995, RMSEA = 0.028, TLI = 0.995, SRMR =0.035, boys: CFI = 0.987, RMSEA = 0.023, TLI = 0.988, SRMR =0.042) and supported overall measurement invariance across pandemic groups.

## **Table S9: Measurement invariance across pandemic groups**

|  | **Model** | **Compared to** | **CFI** | **ΔCFI** | **RMSEA** | **ΔRMSEA** | **TLI** | **SRMR** | **Invariance holds** |
| --- | --- | --- | --- | --- | --- | --- | --- | --- | --- |
| **Girls** |  |  |  |  |  |  |  |  |  |
|  | Configural |  | 0.996 |  | 0.027 |  | 0.995 | 0.029 | Yes |
|  | Threshold | Configural | 0.996 | 0 | 0.027 | 0 | 0.995 | 0.031 | Yes |
|  | Strong | Threshold | 0.995 | -0.001 | 0.029 | 0.002 | 0.995 | 0.033 | Yes |
|  | Strict | Strong | 0.995 | 0 | 0.028 | -0.001 | 0.995 | 0.035 | Yes |
| **Boys** |  |  |  |  |  |  |  |  |  |
|  | Configural |  | 0.991 |  | 0.023 |  | 0.987 | 0.036 | Yes |
|  | Threshold | Configural | 0.990 | -0.001 | 0.023 | 0 | 0.988 | 0.037 | Yes |
|  | Strong | Threshold | 0.988 | -0.002 | 0.023 | 0 | 0.987 | 0.038 | Yes |
|  | Strict | Strong | 0.987 | -0.001 | 0.023 | 0 | 0.988 | 0.042 | Yes |

**Note:** The models tested for measurement invariance were configural, threshold, strong and strict invariance. Invariance held at all steps for both girls and boys, indicating measurement invariance across pandemic groups (pre-pandemic and pandemic). CFI: Comparative fit index; RMSEA: Root mean square error of approximation; SRMR: Standardized root mean square residual; TLI: Tucker-Lewis index.

## **Table S10: Chi-square trend test for each item across pandemic exposure for girls and boys**

|  | **Item** | **Chi-Square** | **df** | **P value** |
| --- | --- | --- | --- | --- |
| **Girls** |  |  |  |  |
|  | Q1 | 56.06 | 1 | **<0.001*** |
|  | Q2 | 28.51 | 1 | **<0.001*** |
|  | Q3 | 42.27 | 1 | **<0.001*** |
|  | Q4 | 20.08 | 1 | **<0.001*** |
|  | Q5 | 2.05 | 1 | 0.15 |
|  | Q6 | 55.37 | 1 | **<0.001*** |
|  | Q7 | 21.35 | 1 | **<0.001*** |
|  | Q8 | 83.07 | 1 | **<0.001*** |
|  | Q9 | 0.22 | 1 | 0.64 |
| **Boys** |  |  |  |  |
|  | Q1 | 12.29 | 1 | **<0.001*** |
|  | Q2 | 26.13 | 1 | **<0.001*** |
|  | Q3 | 7.49 | 1 | **0.006*** |
|  | Q4 | 3.80 | 1 | 0.05 |
|  | Q5 | 0.10 | 1 | 0.76 |
|  | Q6 | 14.40 | 1 | **<0.001*** |
|  | Q7 | 2.44 | 1 | 0.12 |
|  | Q8 | 22.59 | 1 | **<0.001*** |
|  | Q9 | 0.05 | 1 | 0.83 |

**Note:** The table displays the Chi-square statistic, degrees of freedom (df) and p-values for each item (Q1-Q9), assessing the presence of trends in responses from the adolescents (girls and boys) answering before the pandemic and those answering during the pandemic.

## **Figure S3: Mean score for each eating problem item across sex and year of responding to questionnaire**


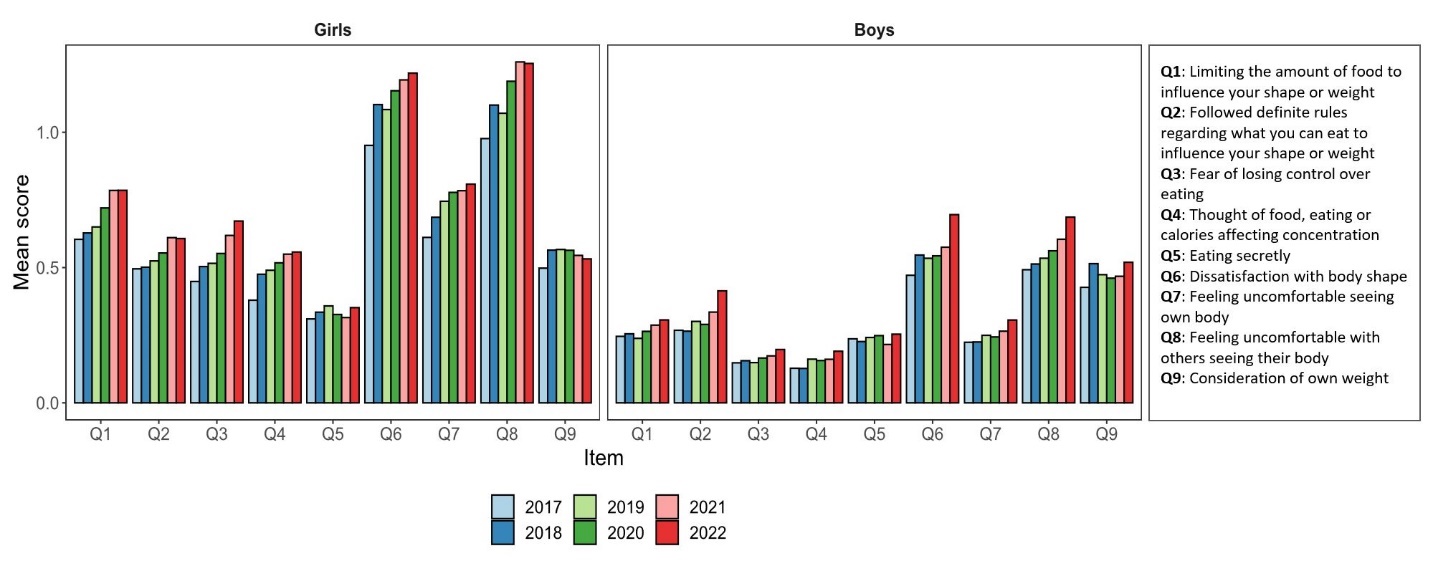


## **Figure S4: Standardized estimates from SEM models of associations between predictors and eating problem domains**

**Note:** 95% confidence intervals are included for all estimates, even where not visible due to high precision.

## **Figure S5: Standardized estimates from SEM models of associations between PGS and eating problem domains**

**Note:** 95% confidence intervals are included for all estimates, even where not visible due to high precision.

## **Table S11: Standardized estimates from SEM models for girls and boys**

| **Factor** | **Covariate** | **Girls** | | **Boys** | |
| --- | --- | --- | --- | --- | --- |
|  |  | **Estimate (SE)** | **P value** | **Estimate (SE)** | **P value** |
| Eating concern and restraint |  |  |  |  |  |
|  | Exercise | 0.038 (0.007) | **<0.001*** | 0.052 (0.008) | **<0.001*** |
|  | Social media | 0.059 (0.009) | **<0.001*** | 0.042 (0.010) | **<0.001*** |
|  | Screen time | -0.002 (0.010) | 0.826 | 0.006 (0.011) | 0.592 |
|  | BMI underweight vs. normal | -0.303 (0.040) | **<0.001*** | -0.119 (0.055) | **0.03** |
|  | BMI overweight vs. normal | 0.434 (0.027) | **<0.001*** | 0.669 (0.031) | **<0.001*** |
|  | Mental distress | 0.551 (0.021) | **<0.001*** | 0.585 (0.032) | **<0.001*** |
|  | Stress | 0.277 (0.017) | **<0.001*** | 0.227 (0.021) | **<0.001*** |
|  | Conflict with parents | 0.195 (0.020) | **<0.001*** | 0.107 (0.027) | **<0.001*** |
|  | Mother’s history of eating disorder/problems | 0.001 (0.057) | 0.986 | 0.148 (0.059) | **0.01*** |
|  | AN PGS | 0.024 (0.010) | **0.017*** | 0.020 (0.012) | 0.098 |
|  | ANX PGS | -0.010 (0.010) | 0.320 | -0.007 (0.012) | 0.552 |
|  | MDD PGS | 0.003 (0.010) | 0.775 | 0.004 (0.013) | 0.780 |
|  | Neuroticism PGS | 0.031 (0.010) | **0.001*** | 0.020 (0.012) | 0.095 |
|  | OCD PGS | 0.019 (0.010) | 0.055 | 0.000 (0.012) | 0.971 |
|  | BMI PGS | 0.057 (0.010) | **<0.001*** | 0.084 (0.012) | **<0.001*** |
|  | Autism PGS | -0.001 (0.010) | 0.887 | 0.011 (0.012) | 0.363 |
| Shape and weight concern |  |  |  |  |  |
|  | Exercise | -0.040 (0.006) | **<0.001*** | -0.071 (0.007) | **<0.001*** |
|  | Social media | 0.041 (0.009) | **<0.001*** | 0.015 (0.009) | 0.108 |
|  | Screen time | 0.039 (0.010) | **<0.001*** | 0.018 (0.010) | 0.076 |
|  | BMI underweight vs. normal | 0.006 (0.034) | 0.855 | 0.208 (0.045) | **<0.001*** |
|  | BMI overweight vs. normal | 0.677 (0.028) | **<0.001*** | 0.604 (0.030) | **<0.001*** |
|  | Mental distress | 0.591 (0.020) | **<0.001*** | 0.678 (0.028) | **<0.001*** |
|  | Stress | 0.365 (0.016) | **<0.001*** | 0.268 (0.018) | **<0.001*** |
|  | Conflict with parents | 0.171 (0.020) | **<0.001*** | 0.146 (0.024) | **<0.001*** |
|  | Mother’s history of eating disorder/problems | 0.043 (0.056) | 0.438 | 0.092 (0.058) | 0.115 |
|  | AN PGS | 0.009 (0.010) | 0.382 | 0.016 (0.011) | 0.149 |
|  | ANX PGS | -0.012(0.010) | 0.215 | 0.001 (0.011) | 0.948 |
|  | MDD PGS | 0.002 (0.010) | 0.825 | -0.001 (0.011) | 0.941 |
|  | Neuroticism PGS | 0.044 (0.010) | **<0.001*** | 0.043 (0.011) | **<0.001*** |
|  | OCD PGS | 0.018 (0.010) | 0.064 | 0.013 (0.011) | 0.213 |
|  | BMI PGS | 0.050 (0.010) | **<0.001*** | 0.009 (0.011) | 0.420 |
|  | Autism PGS | -0.008 (0.010) | 0.426 | -0.015 (0.011) | 0.168 |

**Note:** *Significant after FDR correction

## **Table S12: Model fit comparison between pre-pandemic and pandemic groups across free and constrained parameters among girls**

| **Factor** | **Covariate constrained*** | **F statistic** | **df1** | **df2** | **P value** |
| --- | --- | --- | --- | --- | --- |
| Eating concern and restraint |  |  |  |  |  |
|  | Exercise | 0.216 | 1 | 58425.2 | 0.642 |
|  | Social media | 3.713 | 1 | 76747.7 | 0.053 |
|  | Screen time | 0.050 | 1 | 100687.5 | 0.823 |
|  | BMI underweight vs. normal | 0.282 | 1 | 3223.2 | 0.595 |
|  | BMI overweight vs. normal | 0.196 | 1 | 6160.5 | 0.658 |
|  | Mental distress | 0.150 | 1 | 12204.7 | 0.699 |
|  | Stress | 0.787 | 1 | 16955.1 | 0.375 |
|  | Conflict with parents | 0.996 | 1 | 60205.4 | 0.318 |
|  | Mother’s history of eating disorder/problems | 0.347 | 1 | 93793.0 | 0.556 |
|  | AN PGS | 0.306 | 1 | 1598.7 | 0.580 |
|  | ANX PGS | 1.824 | 1 | 1681.5 | 0.177 |
|  | MDD PGS | 0.223 | 1 | 2060.8 | 0.630 |
|  | Neuroticism PGS | 0.218 | 1 | 3586.7 | 0.641 |
|  | OCD PGS | 0.226 | 1 | 2629.7 | 0.634 |
|  | BMI PGS | 0.231 | 1 | 2759.6 | 0.631 |
|  | Autism PGS | 0.177 | 1 | 3508.1 | 0.674 |
| Shape and weight concern |  |  |  |  |  |
|  | Exercise | 0.253 | 1 | 23524.1 | 0.615 |
|  | Social media | 0.339 | 1 | 31803.5 | 0.561 |
|  | Screen time | 0.533 | 1 | 43761.9 | 0.465 |
|  | BMI underweight vs. normal | 0.493 | 1 | 1469.2 | 0.483 |
|  | BMI overweight vs. normal | 1.146 | 1 | 2256.2 | 0.284 |
|  | Mental distress | 0.405 | 1 | 3570.9 | 0.524 |
|  | Stress | 0.035 | 1 | 83516.9 | 0.851 |
|  | Conflict with parents | 0.0.191 | 1 | 26424.5 | 0.662 |
|  | Mother’s history of eating disorder/problems | 0.098 | 1 | 52377.0 | 0.754 |
|  | AN PGS | 0.287 | 1 | 2812.6 | 0.592 |
|  | ANX PGS | 0.138 | 1 | 4777.6 | 0.710 |
|  | MDD PGS | 2.375 | 1 | 1357.3 | 0.124 |
|  | Neuroticism PGS | 1.113 | 1 | 1381.1 | 0.292 |
|  | OCD PGS | 0.169 | 1 | 4938.0 | 0.681 |
|  | BMI PGS | 0.304 | 1 | 1777.1 | 0.582 |
|  | Autism PGS | 0.182 | 1 | 1748.2 | 0.670 |

**Note:** This table presents F-statistics and corresponding p-values for model fit comparison when covariates are allowed to vary freely (unconstrained) across groups (pre-pandemic and pandemic) versus when they are held to be equal (constrained) across groups. * = Each covariate is constrained one at a time and compared to the unconstrained model.

## **Table S13: Model fit comparison between pre-pandemic and pandemic groups across free and constrained parameters among boys**

| **Factor** | **Model comparison** | **F statistic** | **df1** | **df2** | **P value** |
| --- | --- | --- | --- | --- | --- |
| Eating concern and restraint |  |  |  |  |  |
|  | Exercise | 1.434 | 1 | 23989.1 | 0.231 |
|  | Social media | 0.413 | 1 | 15036.2 | 0.520 |
|  | Screen time | 1.808 | 1 | 15624.3 | 0.179 |
|  | BMI underweight vs. normal | 0.124 | 1 | 5553.6 | 0.725 |
|  | BMI overweight vs. normal | 0.121 | 1 | 10228.5 | 0.728 |
|  | Mental distress | 2.702 | 1 | 4574.9 | 0.100 |
|  | Stress | 0.781 | 1 | 11171.3 | 0.377 |
|  | Conflict with parents | 0.018 | 1 | 377230.8 | 0.893 |
|  | Mother’s history of eating disorder/problems | 0.036 | 1 | 326813.1 | 0.850 |
|  | AN PGS | 0.125 | 1 | 7013.9 | 0.724 |
|  | ANX PGS | 0.767 | 1 | 711.2 | 0.381 |
|  | MDD PGS | 0.440 | 1 | 1642.2 | 0.507 |
|  | Neuroticism PGS | 0.465 | 1 | 1293.8 | 0.495 |
|  | OCD PGS | 0.523 | 1 | 1462.1 | 0.470 |
|  | BMI PGS | 0.244 | 1 | 4932.1 | 0.621 |
|  | Autism PGS | 0.181 | 1 | 4208.3 | 0.670 |
| Shape and weight concern |  |  |  |  |  |
|  | Exercise | 0.717 | 1 | 41688.2 | 0.397 |
|  | Social media | 0.028 | 1 | 280614.4 | 0.868 |
|  | Screen time | 0.028 | 1 | 110183.8 | 0.866 |
|  | BMI underweight vs. normal | 0.175 | 1 | 3326.3 | 0.676 |
|  | BMI overweight vs. normal | 0.884 | 1 | 2737.3 | 0.347 |
|  | Mental distress | 0.079 | 1 | 19825.1 | 0.779 |
|  | Stress | 0.058 | 1 | 44486.8 | 0.810 |
|  | Conflict with parents | 0.412 | 1 | 66021.5 | 0.521 |
|  | Mother’s history of eating disorder/problems | 0.205 | 1 | 121694.6 | 0.651 |
|  | AN PGS | 0.318 | 1 | 2534.8 | 0.573 |
|  | ANX PGS | 0.194 | 1 | 3172.7 | 0.660 |
|  | MDD PGS | 0.761 | 1 | 671.0 | 0.383 |
|  | Neuroticism PGS | 0.324 | 1 | 1876.2 | 0.569 |
|  | OCD PGS | 1.023 | 1 | 882.2 | 0.312 |
|  | BMI PGS | 0.681 | 1 | 1331.8 | 0.410 |
|  | Autism PGS | 0.375 | 1 | 2480.0 | 0.540 |

**Note:** This table presents F-statistics and corresponding p-values for model fit comparison when covariates are allowed to vary freely (unconstrained) across groups (pre-pandemic and pandemic) versus when they are held to be equal (constrained) across groups. * = Each covariate is constrained one at a time and compared to the unconstrained model.

## **Table S14: Model fit indices for SEM and multigroup SEM models**

| **Model** | **Chi square (df)** | **CFI** | **TLI** | **RMSEA** | **SRMR** |
| --- | --- | --- | --- | --- | --- |
| Girls multigroup SEM model (pandemic groups) | 1734.8 (88) | 0.989 | 0.991 | 0.057 | 0.038 |
| Boys multigroup SEM model (pandemic groups) | 996.0 (88) | 0.981 | 0.984 | 0.045 | 0.049 |
| Girls SEM model (with covariates) | 2897.4 (152) | 0.941 | 0.986 | 0.039 | 0.051 |
| Boys SEM model (with covariates) | 1834.0 (152) | 0.903 | 0.977 | 0.032 | 0.058 |
| Girls multigroup SEM model (with covariates and pandemic groups) | 2912.9 (304) | 0.945 | 0.987 | 0.038 | 0.051 |
| Boys multigroup SEM model (with covariates and pandemic groups) | 1934.8 (304) | 0.902 | 0.977 | 0.032 | 0.058 |

**Note**: This table includes model fit indices from all SEM models revealing adequate model fit. CFI: Comparative fit index; RMSEA: Root mean square error of approximation; SRMR: Standardized root mean square residual; TLI: Tucker-Lewis index.

## **Figure S6: Mean score for each predictor across sex and year of responding to questionnaire**


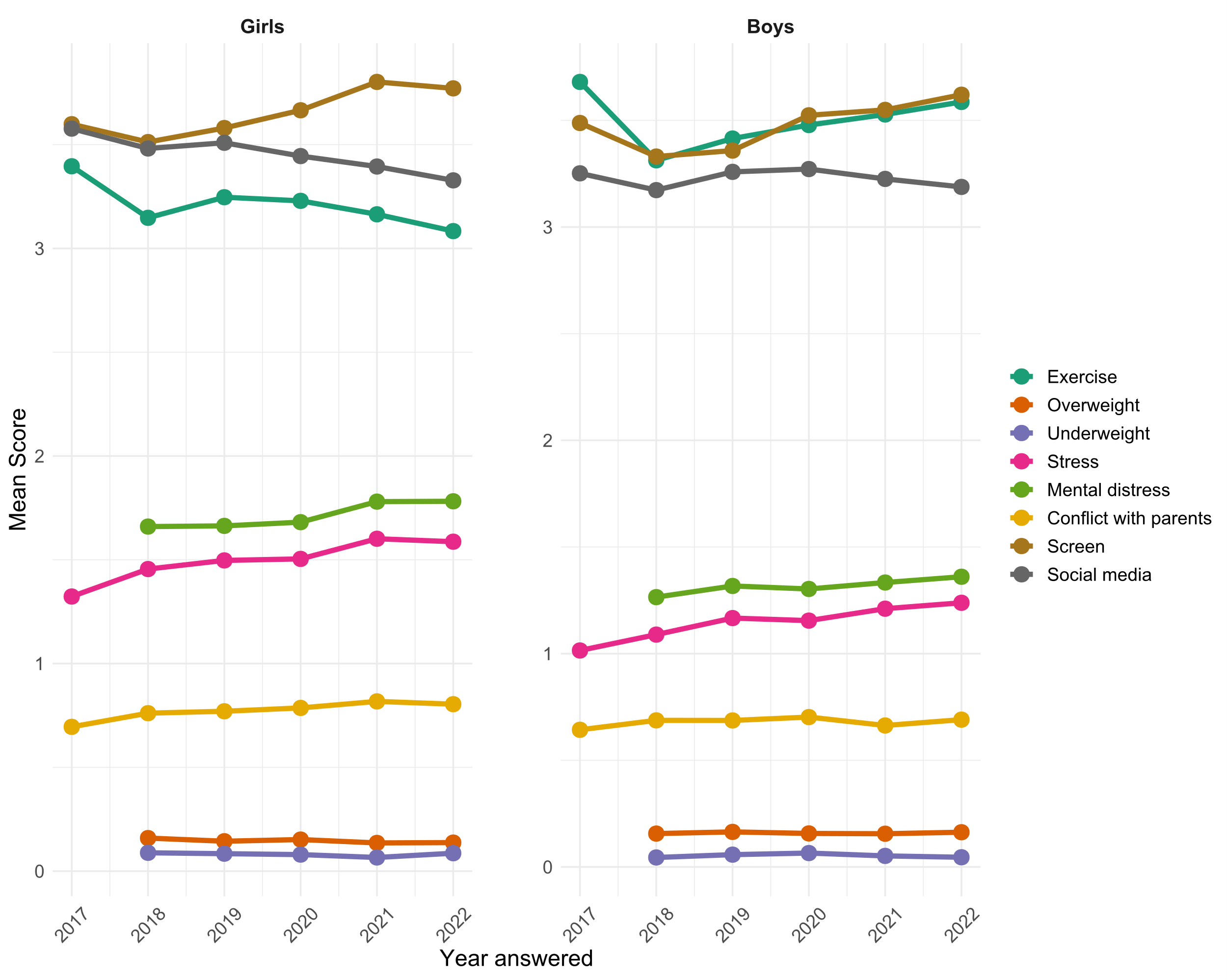


## **Table S15: Mean differences in covariates among adolescents before and during the pandemic**

|  | **Covariate** | **Pre-pandemic Mean (SD)** | **Pandemic Mean (SD)** | **Test statistic** | **P-value** |
| --- | --- | --- | --- | --- | --- |
| **Girls** |  |  |  |  |  |
|  | Exercise | 3.24 (1.48) | 3.18 (1.53) | -2.46 | 0.993 |
|  | Social media communication | 3.50 (1.17) | 3.40 (1.14) | -4.82 | 1 |
|  | Screen time | 3.57 (1.07) | 3.75 (1.02) | 9.64 | **<0.001** |
|  | BMI overweight vs. normal |  |  | 1.27 | 0.259 |
|  | BMI underweight vs. normal |  |  | 1.02 | 0.313 |
|  | Mental distress | 1.66 (0.64) | 1.75 (0.68) | 7.05 | **<0.001** |
|  | Stress | 1.46 (0.81) | 1.56 (0.82) | 7.06 | **<0.001** |
|  | Conflict with parents | 0.75 (0.51) | 0.81 (0.52) | 6.40 | **<0.001** |
| **Boys** |  |  |  |  |  |
|  | Exercise | 3.44 (1.60) | 3.52 (1.63) | 2.43 | **0.008** |
|  | Social media communication | 3.23 (1.21) | 3.24 (1.20) | 0.32 | 0.373 |
|  | Screen time | 3.39 (1.09) | 3.55 (1.10) | 7.54 | **<0.001** |
|  | BMI overweight vs. normal |  |  | 0.55 | 0.457 |
|  | BMI underweight vs. normal |  |  | 0.20 | 0.654 |
|  | Mental distress | 1.30 (0.41) | 1.33 (0.43) | 3.10 | **<0.001** |
|  | Stress | 1.12 (0.69) | 1.19 (0.70) | 5.27 | **<0.001** |
|  | Conflict with parents | 0.68 (0.47) | 0.68 (0.46) | 0.56 | 0.289 |

**Note:** Group differences were evaluated with independent t-tests for all predictors except BMI where we used chi-square trend test. The test statistic column refers to t-value for the t-tests and chi-square value for the chi-square tests.

## **Section 8: Sensitivity analyses**

Girls reported higher overall levels of eating concern/restraint (mean difference = 0.18, SE = 0.04, p<0.001) and shape/weight concern (mean difference=0.20,SE=0.03, p<0.001). For boys, the overall level of eating problems did not differ across pandemic groups.

# **Supplementary references**

1. Willroth EC, Atherton OE. Best laid plans: A guide to reporting preregistration deviations. *Advances in Methods and Practices in Psychological Science*. 2024;7(1):25152459231213802.

2. Alhija F. Factor analysis: An overview and some contemporary advances. *International encyclopedia of education*. 2010;3:162-170.

3. Williams B, Onsman A, Brown T. Exploratory factor analysis: A five-step guide for novices. *Australasian journal of paramedicine*. 2010;8:1-13.

4. Jorgensen TD, Pornprasertmanit, S., Schoemann, A. M., & Rosseel, Y. semTools; Useful tools for structural equation modeling. 2022.

5. Chen FF. Sensitivity of goodness of fit indexes to lack of measurement invariance. *Structural equation modeling: a multidisciplinary journal*. 2007;14(3):464-504.

6. Hannigan LJ, Corfield, E.C., Askelund, A.D., Askeland, R.B., Hegemann, L., Jensen, P., Pettersen, J.H., Rayner, C., Ayorech, Z., Bakken, N.B., Wootton, R., Ask, H., Havdahl, A. phenotools: and R package to facilitate efficient and reproducible use of phenotypic data from MoBa and linked registry sources in the TSD environment. . 2023;doi:10.17605/OSF.IO/6G8BJ

7. Choi SW, O'Reilly PF. PRSice-2: Polygenic Risk Score software for biobank-scale data. *Gigascience*. Jul 1 2019;8(7)doi:10.1093/gigascience/giz082

8. Coombes BJ, Ploner A, Bergen SE, Biernacka JM. A principal component approach to improve association testing with polygenic risk scores. *Genet Epidemiol*. 2020;44(7):676-686.
